# Analyzing Coarsened and Missing Data by Imputation Methods

**DOI:** 10.1002/sim.70032

**Published:** 2025-03-05

**Authors:** Lars L. J. van der Burg, Stefan Böhringer, Jonathan W. Bartlett, Tjalling Bosse, Nanda Horeweg, Liesbeth C. de Wreede, Hein Putter

**Affiliations:** ^1^ Biomedical Data Sciences Leiden University Medical Center Leiden The Netherlands; ^2^ London School of Hygiene and Tropical Medicine London UK; ^3^ Department of Pathology Leiden University Medical Center Leiden The Netherlands; ^4^ Department of Radiation Oncology Leiden University Medical Center Leiden The Netherlands; ^5^ DKMS Dresden/Tübingen Germany; ^6^ Mathematical Institute Leiden University Leiden The Netherlands

## Abstract

In various missing data problems, values are not entirely missing, but are coarsened. For coarsened observations, instead of observing the true value, a subset of values ‐ strictly smaller than the full sample space of the variable ‐ is observed to which the true value belongs. In our motivating example for patients with endometrial carcinoma, the degree of lymphovascular space invasion (LVSI) can be either absent, focally present, or substantially present. For a subset of individuals, however, LVSI is reported as being present, which includes both non‐absent options. In the analysis of such a dataset, difficulties arise when coarsened observations are to be used in an imputation procedure. To our knowledge, no clear‐cut method has been described in the literature on how to handle an observed subset of values, and treating them as entirely missing could lead to biased estimates. Therefore, in this paper, we evaluated the best strategy to deal with coarsened and missing data in multiple imputation. We tested a number of plausible ad hoc approaches, possibly already in use by statisticians. Additionally, we propose a principled approach to this problem, consisting of an adaptation of the SMC‐FCS algorithm (SMC‐FCS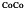: Coarsening compatible), that ensures that imputed values adhere to the coarsening information. These methods were compared in a simulation study. This comparison shows that methods that prevent imputations of incompatible values, like the SMC‐FCS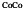 method, perform consistently better in terms of a lower bias and RMSE, and achieve better coverage than methods that ignore coarsening or handle it in a more naïve way. The analysis of the motivating example shows that the way the coarsening information is handled can matter substantially, leading to different conclusions across methods. Overall, our proposed SMC‐FCS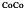 method outperforms other methods in handling coarsened data, requires limited additional computation cost and is easily extendable to other scenarios.

## 1 Introduction

A frequent problem in statistical analyses is the presence of incomplete data. In the most commonly studied setting, the observation of one or more variables is completely unknown (missing) for a subset of individuals. For this setting, an extensive “missing data” framework has been derived, with approaches for different missingness assumptions [1, 2]. When the missingness is assumed to be missing at random (MAR) ‐ meaning that the probability of a value to be missing only depends on observed data ‐ multiple imputation (MI) can be applied to deal with the missing observations to obtain valid inference. In this three‐step approach, missing data is iteratively drawn from an imputation model forming several complete data sets, which are then analyzed as usual via a regression model or another standard approach. Results from these regression models are pooled to obtain estimates of the parameters of interest. It is important for valid inference that the imputation and regression model of the first two steps are compatible, which might be challenging for a non‐linear relationship between predictors and outcome. The substantive model compatible fully conditional specification (SMC‐FCS) procedure ensures compatibility by accommodating the substantive model for the outcome in the imputation procedure [3].

However, in various common situations incomplete data is not entirely missing. Instead, the value of the variable for a subject is only partly unknown; it is known that the value belongs to a strict subset of the sample space of the variable. This phenomenon has been termed coarsening [4], and the resulting data are referred to as coarsened data. This name is used as an overarching term for various forms of partly observed data, including censoring and grouping of data [5, 6]. Censoring denotes the situation when only a lower or upper bound for a value is known, such as in survival analysis, when the occurrence of an event takes place outside the observational window and only minimal event‐free time can be defined as end of follow‐up. Within the field of survival analysis, multiple methods have been specifically designed to account for censoring and to obtain valid estimates. However, problems arise when instead of a censored outcome, one of the covariates is censored, for example, when the age of clinical diagnosis is an important predictor but this time point lies after the observational period [7]. Examples of grouping are the categorization of a continuous age into various intervals, or grouping multiple disease categories into a single level. Grouping can be intentional to reduce model complexity, but can also arise due to limitations in the measurement process. For example, limitations in the *KIR* genotyping process often lead to ambiguous genotype calls, each corresponding to a subset of compatible diplotypes [8]. These subsets exclude already a large number of diplotypes, but the true diplotype remains unknown.

Similar to the missingness framework, there are different coarsening mechanisms, which make different assumptions about the coarsening mechanism given observed data [9]. For coarsening completely at random (CCAR) the coarsening occurs randomly, independently of observed data, leading to unbiased results when a complete case analysis, where both completely missing or coarsened observations are discarded, is employed [10]. The coarsening at random (CAR) assumption is similar to the MAR assumption in the sense that both assume that the probability of a particular coarsened observation occurring depends only on observed information. Intuitively, the CAR assumption implies that each possible value within the subset has the same probability of becoming coarsened, conditional on other observed variables. A formal definition will be given in the next section. With coarsening not at random (CNAR), which implies that coarsening depends also on unobserved information, the problem becomes non‐identifiable.

The motivation for this paper is a study in endometrial cancer, where data from a number of clinical trials were combined for a model predicting recurrence. An important risk factor for recurrence in this disease is lymphovascular space invasion (LVSI) ‐ indicating to what degree the cancer has spread into the blood and lymph vessels of the myometrium. Commonly, this variable is scored as either absent, focally present or substantially present [11]. However, in a number of trials the variable was only recorded to be either absent or present, where the present group is a combination of focally and substantially present. Since only the substantial presence of LVSI has been shown to be associated with recurrence, interest lies in the use of all three levels [12]. Thus, the observations for the individuals recorded as present are coarsened and need to be handled appropriately.

In this paper, we investigate the best strategy to handle such coarsened data occurring in categorical covariates with multiple imputation procedures. We work in a context where the covariates are used in a regression model and the main interest is in the regression parameters. To our knowledge, no thorough investigation is available for such a data problem. In Section 2 we propose a principled method based on an adaptation of the substantive model compatible fully conditional specification (SMC‐FCS), and suggest a number of intuitive ad hoc approaches, which are tested in a simulation study in Section 3. Section 4 illustrates the methods on the motivating dataset, and the paper closes with a discussion in Section 5.

## 2 Methodology

Our main contribution is an extension of the SMC‐FCS approach to MI to accommodate coarsened data in Section 2.1. We first briefly review SMC‐FCS, motivate our proposal to incorporate coarsened data in this setting, and discuss how the {smcfcs} package in R can be extended to include coarsened data. We then restrict to categorical coarsened data and review some intuitive ad hoc approaches in Section 2.2.

### 2.1 Coarsening Compatible SMC‐FCS

Bartlett et al. [3] consider the setting where a substantive model, that is, a model representing a research question, for a fully observed outcome Y has been specified based on covariates, of which some are partially observed, $X=(X_{1},\ldots ,X_{p})$, and the remainder are fully observed, $Z=(Z_{1},\ldots ,Z_{q})$. In the standard missing data setting, an individual observation in one of the partially observed covariates is either completely missing or present. The indicator value R of this observation then takes the values 0 (when missing) or 1 (when present). Letting $X_{\text{obs}}$ and $X_{\text{mis}}$ denote the observed and missing components of X for a given individual, the MAR assumption states that $P(R\;|\;X,Y,Z)=P(R\;|\;X_{\text{obs}},X_{\text{mis}},Y,Z)=P(R\;|\;X_{\text{obs}},Y,Z)$.

Coarsening can be defined by allowing set‐valued observations for each partially observed covariate $X_{j}$. For the corresponding sampling space $\Omega_{j}$, an individual has observation $X_{j}^{\prime }\subset \Omega_{j}$. If $X_{j}^{\prime }=\Omega_{j}$, then $X_{j}$ is completely missing, while if $X_{j}^{\prime }$ is a single point, $X_{j}$ is completely observed for the respective subject. Otherwise, an observation is said to be properly coarsened for any other subset $X_{j}^{\prime }$. Therefore, $X_{j}^{\prime }\in 𝒫(\Omega_{j})$, the *power set* of $\Omega_{j}$, which is finite if and only if $X_{j}$ is discrete. In practice, also if the distribution of $X_{j}$ is continuous, in a finite sample only a finite number of possible subsets of $\Omega_{j}$ will be observed. To simplify the exposition, we assume that $X_{j}^{\prime }$ can only take a finite number of values (non‐empty subsets of $\Omega_{j}$). We enumerate all possible distinct combinations of observations of $X_{j}^{\prime }$, and categorize all (observed) subsets of Ω as 0 (completely missing), 1 (completely observed), $2,\ldots ,{ℛ}_{j}$, where ${ℛ}_{j}$ is the total number of these combinations. Let $R=(R_{1},\ldots ,R_{J})$ be a vector of coarsening indicators $R_{j}$, with $R_{j}\in \{0,\ldots ,{ℛ}_{j}\}$. We extend the original definition of R used to distinguish between MCAR, MAR and MNAR, to include these possible values, thus changing the definition of $R_{j}$ from a missing data indicator to a coarsening indicator. The *coarsening at random* (CAR) assumption now states that $P(R\;|\;X,Y,Z)=P(R\;|\;X^{\prime },Y,Z)$, both being multinomial distributions. Tsiatis [10] gives an equivalent definition.

The substantive model is denoted by f(Y|X,Z;ψ) with parameter ψ, which we assume to be well specified. In fully conditional specification multiple imputation (FCS MI) ‐ the procedure underlying the MICE algorithm ‐ models are specified for each partially observed variable, conditional on all other variables and the outcome. Denote the chosen model for partially observed covariate $X_{j}$ by $f(X_{j}\;|\;X_{-j},Z,Y,\theta_{j})$, parameterized by $\theta_{j}$. Typically, a generalized linear model is used, which, in general, is not compatible with the substantive model. FCS MI starts by replacing missing values in each $X_{j}$ by observed values from $X_{j}$. Then missing values are repeatedly imputed, conditioning on the most recent imputed values of the other variables. Define $x_{j}^{\text{mis},(t)}$ to be the imputed values of $x_{j}^{\text{mis}}$ in iteration t, $x_{j}^{\left(t\right)}=(x_{j}^{\text{obs}},x_{j}^{\text{mis},(t)})$ the completed vector of observed and imputed values at iteration t, and $x_{-j}^{\left(t\right)}=(x_{1}^{\left(t\right)},\ldots ,x_{j-1}^{\left(t\right)},x_{j+1}^{\left(t-1\right)},\ldots ,x_{p}^{\left(t-1\right)})$. Then the $t^{\text{th}}$ iteration of FCS MI consists of drawing, first from $\theta_{j}^{\left(t\right)}∼f(\theta_{j})f(x_{j}^{\text{obs}}\;|\;x_{-j}^{\left(t\right)},z,y,\theta_{j})$, j=1,…,p, then from $x_{j}^{\text{mis},(t)}∼f(x_{j}^{\text{mis}}\;|\;x_{-j}^{\left(t\right)},z,y,\theta_{j}^{\left(t\right)})$. Above steps are continued until convergence, although in practice, a finite number of iteration steps is chosen. This whole procedure is repeated m times, where each time the last iteration is taken, forming m imputed datasets. Each imputed dataset is analyzed with the same substantive regression model and coefficients are pooled with Rubin's rules [1, 2]. This procedure is regularly used, and has been implemented in the R package {mice}.

The idea behind SMC‐FCS is to specify an imputation model for $X_{j}$ that is compatible with the substantive model. Models $f(X_{j}\;|\;X_{-j},Z,\phi_{j})$ are specified, with non‐informative priors for the parameters ψ and $\phi_{j}$. Then, noting that

(1) $$f(X_{j}\;|\;X_{-j},Z,Y)\propto f(Y\;|\;X,Z)f(X_{j}\;|\;X_{-j},Z)$$

the idea is to impute missing values in $X_{j}$ from the density proportional to the product of f(Y|X,Z,ψ) and $f(X_{j}\;|\;X_{-j},Z,\phi_{j})$. This product density typically does not belong to a standard class of parametric distributions, requiring sampling methods such as rejection sampling to draw observations [3]. Note that for discrete variables direct draws are possible, since then for each possible value $x_{j}\in \Omega_{j}$ the product of $P(X_{j}=x_{j}\;|\;X_{-j},Z,\phi_{j})$ and f(Y|X,Z,ψ) (X containing $x_{j}$) can be calculated, their standardized (so as to add up to one) values defining a multinomial distribution on $\Omega_{j}$. For the $t^{\text{th}}$ iteration, these probabilities are calculated by first drawing $\psi^{\left(t,j\right)}∼f(\psi )f(y\;|\;x_{j}^{\left(t-1\right)},x_{-j}^{\left(t\right)},z,\psi )$ and $\phi_{j}^{\left(t\right)}∼f(\phi_{j})f(x_{j}^{\left(t-1\right)}\;|\;x_{-j}^{\left(t\right)},z,\phi_{j})$, j=1,…,p, and then drawing the missing values of $X_{j}$ from the density proportional to (1) [3]. This way, in each iteration, the imputed values for the missing values of $X_{j}$ are updated. This is repeated for a pre‐specified number of iterations, where all iterations up to (and not including) t (here t=20) are used as “burn‐in” samples to reach convergence of the sampler.

Suppose now that for a particular subject the value of $X_{j}$ is properly coarsened and suppose $X_{j}^{\prime }=S$ is observed, in other words it is known that only the points in subset S of $\Omega_{j}$ are compatible with $X_{j}$. If the above SMC‐FCS approach is naïvely applied by ignoring the coarsening information, imputations will be drawn under the assumption that $X_{j}$ is completely missing, possibly leading to imputations outside S. Since we know that $X_{j}\in S$, the aim is to impute from $f(X_{j}\;|\;X_{j}\in S,X_{-j},Z,Y)$, rather than from $f(X_{j}\;|\;X_{-j},Z,Y)$, as would be the case if $X_{j}$ is completely missing for the individual. Note that by the CAR assumption

(2) $$f(X_{j}\;|\;X_{j}\in S,X_{-j},Z,Y)=\left\{\begin{array}{ll} 0,\; & \text{if}\;X_{j}\notin S;\\ \frac{f(X_{j}\;|\;X_{-j},Z,Y)}{P(X_{j}\in S\;|\;X_{-j},Z,Y)},\; & \text{if}\;X_{j}\in S \end{array}\right.$$

which is proportional to $f(Y\;|\;X,Z)f(X_{j}\;|\;X_{-j},Z)$, as in Equation (1), *but limited to* $X_{j}\in S$. Whereas, SMC‐FCS allows to impute missing values in $X_{j}$ from the density proportional to $f(Y\;|\;X,Z,\psi )f(X_{j}\;|\;X_{-j},Z,\phi_{j})$, then the coarsening compatible SMC‐FCS (SMC‐FCS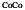) method simply adds a (second) rejection step, which accepts a value $X_{j}$ drawn using SMC‐FCS only if $X_{j}\in S$, and rejects it otherwise. Since $X_{j}$ is discrete, again it is possible to avoid rejection sampling by first calculating the product of $P(X_{j}=x_{j}\;|\;X_{-j},Z,\phi_{j})$ and f(Y|X,Z,ψ) for all values $x_{j}$ limited to $x_{j}\in S$. Subsequently, values for $X_{j}\in S$ can be drawn with probabilities relative to these calculated values. In practice, this means that when $X_{j}$ is discrete, two adjustments are made to the SMC‐FCS algorithm: (1) initial values for the missing values in each $X_{j}$ are drawn from the set of observed values that are compatible with S, and (2) probabilities estimated for points that are not compatible with S are set to zero before normalization of the probabilities. The implementation of this extension in the SMC‐FCS package is described in Appendix A.

### 2.2 Other Methods

The SMC‐FCS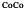 method is generally applicable whenever SMC‐FCS can be used. The simulation study in Section 3 illustrates SMC‐FCS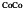 for the special case of a single categorical covariate with coarsening and potential missingness. The reason for that is that for this common situation, other ad hoc methods seem obvious to suggest and are used for method comparison. For the remainder of the paper we restrict to this case.

Our motivating example, mentioned in the introduction and further detailed in Section 4, concerns the PORTEC and MST studies in which the variable LVSI was assessed through a central pathology review [12]. In PORTEC‐1, PORTEC‐2 and MST, the variable LVSI is quantified in three levels: Absent, focally present and substantially present [13, 14, 15]. However, for a subset of patients, the coarsened present, meaning either focally or substantially present, is also observed. In the PORTEC‐3 trial, the LVSI variable was registered dichotomized, as absent or coarsened present [16]. We use this setting to guide the simulation study in the next section, and also to explain the other methods to be compared in the simulation study now. To simplify the notation but without loss of generality, we use a variable X with three levels: a, b and c. The only coarsening we consider for a subset of the observations X is that the observation is either b or c, which is indicated by {b,c} (present), rather than b or c. For another subset of observations, X is completely missing. Table 1 shows data for 8 patients, the first three of which are completely observed, the next two are coarsened, and the last three are missing. Column $X_{\text{compl}}$ shows the true (but unobserved) information. The information that is observed is shown in column $X_{\text{obs}}$, which can take values a, b, c, {b,c} (so the coarsened combination of b and c) or NA (completely missing). For some of the ad hoc methods, the observed information is split into two columns for further analysis, X and C, which will then be considered as the partially missing covariates in the multiple imputation procedure. Column X contains the “certain” information of $X_{\text{obs}}$, that is, it copies the observations that are completely observed, and is missing otherwise. The coarsening column C takes values a, {b,c}, or is completely missing (which can be denoted as NA or as {a,b,c}). The idea behind introducing the auxiliary covariate C is that during the imputation cycles information about X, needed for instance for individuals 4 and 5 in Table 1, can be borrowed from C, for instance from individuals 2 and 3. Columns $Z_{1}$, $Z_{2}$ and Y in Table 1 are two fully observed covariates and the outcome, respectively.

**TABLE 1:** Coarsening data example for nine individuals. For individuals 1–3 X is completely observed, for individuals 4 and 5 X is in a situation with coarsening, indicated by {b,c} the coarsened combination of b or c, and for individuals 6–8 X is missing. Column $X_{\text{compl}}$ contains the true (but unobserved) measurement, column $X_{\text{obs}}$ the observed information, which is split into two columns X and C. Column C is differently structured (NA or {a,b,c}) for different methods. Lastly, columns $Z_{1}$, $Z_{2}$ and Y are two fully observed covariates and the outcome.

ID	$X_{\text{compl}}$	$X_{\text{obs}}$	X	C	$Z_{1}$	$Z_{2}$	Y
1	a	a	a	a	−0.966	−0.166	−0.170
2	b	b	b	{b,c}	1.097	−0.619	1.592
3	c	c	c	{b,c}	0.714	2.389	4.592
4	b	{b,c}	NA	{b,c}	−0.291	0.743	−0.543
5	c	{b,c}	NA	{b,c}	0.729	0.456	2.270
6	a	NA	NA	NA/{a,b,c}	1.035	0.204	1.387
7	b	NA	NA	NA/{a,b,c}	−0.351	−0.317	0.637
8	c	NA	NA	NA/{a,b,c}	0.224	−0.150	0.430

The set‐up described above is thus based on the above mentioned PORTEC and MST studies, but other options for coarsening (e.g., {a,b} or {a,c}) would be equally possible. With multiple ways of coarsening $X_{\text{compl}}$, multiple auxiliary C columns would be needed.

To deal with the coarsened data, we consider several methods. Not all methods are expected to perform equally well, but they are chosen because of their simplicity and therefore their expected use in practice. For all methods, the imputation model for X depends on the outcome Y and the auxiliary variables $Z_{1}$ and $Z_{2}$. The methods differ in the way they structure and handle the coarsening variable C. In contrast, the substantive model for each method is the same: The outcome is regressed on X, $Z_{1}$ and $Z_{2}$, while C is ignored (but information in C is possibly indirectly transferred into X through the imputation model). Since it is possible to have a dataset where observations can be either coarsened or completely missing, all methods are equipped to handle both under the assumptions of CAR and MAR.

1. Complete case analysis (CCA): All individuals with a coarsened or missing observation are discarded from the dataset.
2. MICE: Multiple imputation via the MICE algorithm [17] (R package {mice}): The missing values in X are imputed via the multinomial logistic regression (“polytomous”) regression for unordered categorical data (“polyreg”) approach. Four different sub‐approaches are used.
  – MICE: C is ignored in the imputation model.
  – MICE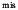: C is included in the imputation model as extra auxiliary variable. When there are also missing observations in X, these individuals will also have missing observations in C. These missing observations in C will be denoted by NA (Table 1), and will thus also be imputed via an imputation model that depends on X, $Z_{1}$, $Z_{2}$ and Y via the “polyreg” approach.
  – MICE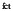: C is included in the imputation model as extra auxiliary variable. When there are also completely missing observations in X, these missing observations in C will be grouped in the separate factor level {a,b,c} (Table 1) and thus do not need to be imputed. Thus, the NA's in column C of Table 1 are considered a separate category. When no missing observations are present in X, MICE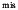 and MICE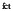 are equivalent.
  – MICE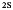: Two‐step approach. In step 1 a subset is made containing all individuals with coarsened observations (i.e., all observations with {b,c}) and with fully observed b or c. The coarsened observations in this subset are then imputed based only on observations that are b or c to ensure compatibility. When there are also missing observations in X, these are imputed in step 2 based on a completed dataset, containing the imputed coarsened observations of step 1 and any additional missing observations. Without missing observations in X, the second step is omitted. To end up with the same number of imputed datasets as in the other methods, in step 2 only one dataset is imputed for each imputed dataset from step 1.
3. SMC‐FCS: Imputations via the default SMC‐FCS algorithm [18], as implemented in the {smcfcs} package in R. The same four approaches are studied as for the MICE algorithm, named SMC‐FCS, SMC‐FCS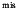, SMC‐FCS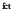 and SMC‐FCS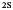. Here the missing values in X, and where applicable C, are imputed via the multinomial logistic regression for unordered categorical variables (“mlogit”) approach.
4. SMC‐FCS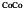: Imputations via the SMC‐FCS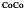 algorithm, as described in Section 2.1.

## 3 Simulation Study

### 3.1 Set‐Up

The simulation studies described here follow the aims, data‐generating mechanisms, estimands, methods, and performance measures (ADEMP)‐structure discussed in Morris et al. [19].

#### 3.1.1 Aim

The aim of the simulation study is to evaluate SMC‐FCS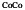 and the methods suggested in Section 2.2, to deal with coarsened and missing data under different settings.

#### 3.1.2 Data Generation Mechanism

For each dataset, $n_{\text{obs}}=2000$ individuals were simulated, each with three explanatory variables ($X,Z_{1}$ and $Z_{2}$) and an outcome (Y). Covariate X is categorical with three levels, denoted a, b, and c, while $Z_{1}$ and $Z_{2}$ are continuous. For each scenario described below, $n_{\text{sim}}=165$ independent replications were run. This number is based on the desired Monte‐Carlo Standard Error (MCSE) of the bias of all regression coefficients, which is defined as $\text{MCSE(bias)}=\sqrt{\sigma^{2}/n_{\text{sim}}}$. For this study, a MCSE(bias)≤0.01 was deemed to be acceptable. To determine the $\sigma^{2}$ per parameter, a pilot study was run with different scenarios. A global $\sigma^{2}$ was chosen as the 95% percentile over all estimated $\sigma^{2}s$, which was found to be 0.128. This led to a final sample size ($n_{\text{sim}}$) of 165.

*Covariates*

Three covariates $\left(\tilde{X},Z_{1},Z_{2}\right)$ were simulated following a trivariate normal distribution with mean μ=(0,0,0), and variance‐covariance matrix ∑ with diagonal elements $\sigma_{\tilde{X}}^{2}=\sigma_{Z_{1}}^{2}=\sigma_{Z_{2}}^{2}=1$ and correlations $\rho_{\tilde{X}Z_{1}}=\rho_{\tilde{X}Z_{2}}=\rho_{Z_{1}Z_{2}}=0$. To investigate performance under different scenarios, we also evaluated alternative choices $\rho_{\tilde{X}Z_{1}}=0.7$, $\rho_{\tilde{X}Z_{2}}=0.3$ and $\rho_{Z_{1}Z_{2}}=0.7$, leading to a total of 8 sets of correlation parameters. A categorical variable X with $P(X=a)=p_{a}$, $P(X=b)=p_{b}$, and $P(X=c)=p_{c}$, with $p_{a}+p_{b}+p_{c}=1$ was derived by dividing $\tilde{X}$ into three disjunct intervals, where $\tilde{X}\leq \Phi^{-1}(p_{a})$ corresponds to X=a, $\Phi^{-1}(p_{a})<\tilde{X}\leq \Phi^{-1}(p_{a}+p_{b})$ corresponds to X=b, and $\tilde{X}>\Phi^{-1}(p_{a}+p_{b})$ corresponds to X=c (Φ(x) representing the cumulative distribution function of a standard normal random variable, see Bonneville et al. [20]). We considered the following values for $\left(p_{a},p_{b},p_{c}\right)$: $\left(\frac{1}{3},\frac{1}{3},\frac{1}{3}\right)$, $\left(\frac{1}{2},\frac{1}{4},\frac{1}{4}\right)$, and $\left(\frac{1}{2},\frac{1}{3},\frac{1}{6}\right)$. In the simulation study, we refer to these values as uniform, bc‐uniform, and skewed, respectively.

*Outcomes*

It is well known that SMC‐FCS performs similarly to MICE for continuous (normal) outcomes with covariates entered linearly, but outperforms MICE for non‐linear substantive models, like logistic or Cox regression [3]. For this reason, we consider both a continuous, normally distributed, outcome and a time‐to‐event outcome. A continuous outcome was drawn according to

(3) $$\begin{array}{l} Y=\beta_{0}+\beta_{1}\text{1}\{X=b\}+\beta_{2}\text{1}\{X=c\}+\beta_{3}Z_{1}+\beta_{4}Z_{2}+\epsilon \end{array}$$

where $\epsilon_{i}\overset{i.i.d.}{∼}N(0,1)$. A survival outcome was simulated as

(4) $$\begin{array}{ll} & \tilde{T}∼\exp(\exp\{\beta_{0}+\beta_{1}\text{1}\{X=b\}+\beta_{2}\text{1}\{X=c\}+\beta_{3}Z_{1}+\beta_{4}Z_{2}\})\\ & T_{C}∼\text{Unif}(5,10) \end{array}$$

the model has a constant baseline hazard and the $\tilde{T}$ and $T_{C}$ contain an event time and a censoring time for each individual, respectively. We define $T:=\min(\tilde{T},T_{C})$ as the individual's observed time, with corresponding event indicator $D:=\text{1}(\tilde{T}\leq T_{C})$.

Reference values of the regression coefficients were taken to be $\beta_{0}=0$ for the continuous outcome and $\beta_{0}=\log(0.1)$ for the survival outcome, $\beta_{1}=\beta_{3}=\beta_{4}=0.5$ and $\beta_{2}=1$. Moreover, additional scenarios were generated by multiplying $\beta_{1}$ and $\beta_{2}$ by {0.5,1,2} (effect sizes).

*Coarsening and Missing Data*

Coarsening and missingness was induced only in X, meaning that both $Z_{1}$, $Z_{2}$ and the outcome were always completely observed. We induced coarsening and missingness in X, depending on $Z_{1}$ and $Z_{2}$, using a multinomial logistic regression set‐up for a random variable R taking the values 0 (X completely missing), 1 (X completely observed) and 2 (coarsening in X), with probabilities

$$\begin{array}{ll} P(R=0\;|\;Z) & =\frac{1}{1+e^{\gamma_{1}^{⊺}Z}+e^{\gamma_{2}^{⊺}Z}},\\ P(R=r\;|\;Z) & =\frac{e^{\gamma_{r}^{⊺}Z}}{1+e^{\gamma_{1}^{⊺}Z}+e^{\gamma_{2}^{⊺}Z}},\;r=1,2 \end{array}$$

with $Z=(1,Z_{1},Z_{2})$. Default values for $\gamma_{1}$ and $\gamma_{2}$ were $\gamma_{1}=(\gamma_{10},\gamma_{11}=1,\gamma_{12}=0)$ and $\gamma_{2}=(\gamma_{20},\gamma_{21}=0,\gamma_{22}=1)$, where $\gamma_{10}$ and $\gamma_{20}$ were chosen so that pre‐specified percentages of coarsening and missingness were obtained, namely (P(R=0),P(R=1),P(R=2))=(0.0,0.4,0.6) and (0.2,0.4,0.4).

Coarsening could only apply to observations for which X=b or X=c, so for observations with X=a coarsening was not applied. Observations that were coarsened or missing were both made missing in X, where a coarsening indicator C was created to distinguish between these two, which is defined as

(5) $$\begin{array}{l} C=\left\{\begin{array}{ll} a, & \text{if}\;X=a\;\&\;R\neq 0\\ \{b,c\}, & \text{if}\;(X=b\;\text{or}\;X=c)\;\&\;R\neq 0\\ \text{NA}, & \text{if}\;R=0 \end{array}\right. \end{array}$$

#### 3.1.3 Design

The simulation study follows a full factorial design, where the parameter sets mentioned above are evaluated in all combinations. Two combinations of the correlation parameters, namely the scenarios with $\rho_{\tilde{X}Z_{1}}=0$, $\rho_{\tilde{X}Z_{2}}=0.3$, and $\rho_{Z_{1}Z_{2}}=0$ or $\rho_{Z_{1}Z_{2}}=0.7$, were not investigated. This results in 6 (correlation parameters) × 3 (category frequencies, choices for $\left(p_{a},p_{b},p_{c}\right)$) × 2 (outcomes) × 3 (effect sizes) × 2 (coarsening strength, γ's) = 216 combinations.

#### 3.1.4 Estimands

The main estimand of the simulation studies is the vector of regression coefficients $\left(\beta_{0},\beta_{1},\ldots ,\beta_{4}\right)$ of intercept (only linear regression), indicators of X, and of $Z_{1}$ and $Z_{2}$. A second estimand is the percentage correct classifications of the coarsened individuals.

#### 3.1.5 Methods to Evaluate

Each simulated dataset was analyzed with the methods described in Sections 2.1 and 2.2.

All missing observations were imputed based on the observed auxiliary covariates $Z_{1}$ and $Z_{2}$ and the outcome, and depending on the method, based on C. When a survival outcome was simulated, the MICE algorithm included the Nelson‐Aalen estimate of the marginal cumulative hazard and the event indicator as outcome information [21]. Default imputation models were used for each imputation approach. For each simulated dataset, 50 imputed datasets are made. Each imputed dataset was analyzed with the same analysis model, namely a well‐specified linear regression model or a Cox proportional hazards model with X (categorical), $Z_{1}$ and $Z_{2}$ (linear) as covariates.

#### 3.1.6 Performance Measures

For each of the regression coefficients β in our substantive regression model, each method yields ${\hat{\beta }}_{i},i=1,\ldots ,n_{\text{sim}}$, as the estimates, and ${\hat{\text{SE}}}_{i},i=1,\ldots ,n_{\text{sim}}$, as the estimated standard errors for the ith replication. Based on these, define the averages $\bar{\hat{\beta }}=\frac{1}{n_{\text{sim}}}\sum_{m=1}^{n_{\text{sim}}}{\hat{\beta }}_{i}$ and $\bar{\hat{\text{SE}}}=\frac{1}{n_{\text{sim}}}\sum_{i=1}^{n_{\text{sim}}}{\hat{\text{SE}}}_{i}$. With these coefficients, the root mean square error (RMSE) $=\sqrt{\frac{1}{n_{\text{sim}}}\sum_{i=1}^{n_{\text{sim}}}{\left({\hat{\beta }}_{i}-\beta \right)}^{2}}$, bias $=\bar{\hat{\beta }}-\beta$ and the coverage $=\frac{1}{n_{\text{sim}}}\sum_{i=1}^{n_{\text{sim}}}\text{1}\{{\hat{\beta }}_{i}-z_{0.975}{\hat{\text{SE}}}_{i}<\beta <{\hat{\beta }}_{i}+z_{0.975}{\hat{\text{SE}}}_{i}\}$ are calculated as performance measures.

Another performance measure that we consider is the *percentage incompatibly classified coarsened individuals*. Considering the individuals with a coarsened observation, that is, with an $X_{\text{obs}}=\{b,c\}$, we calculate the percentage of observations that are imputed incompatibly with the coarsened observation, so that are imputed with an a, while the observation is either an b or an c. This percentage is shown as an average over all imputed datasets over all replications.

### 3.2 Simulation Results

For clarity of exposition, the 216 simulation combinations are divided into 12 scenarios, each with 18 sub‐scenarios. The scenarios are divided based on the outcome (continuous: Scenarios 1–6 and survival: Scenarios 7–12) and correlation structure between X, $Z_{1}$ and $Z_{2}$ (no correlation: Scenarios 1 and 7; correlation between X and $Z_{1}$ only: Scenarios 2 and 8; correlation between X and $Z_{1}$ and between X and $Z_{2}$, but no correlation between $Z_{1}$ and $Z_{2}$: Scenarios 3 and 9; correlation between $Z_{1}$ and $Z_{2}$ only: Scenarios 4 and 10; correlation between X and $Z_{1}$ and between $Z_{1}$ and $Z_{2}$, but no correlation between X and $Z_{2}$: Scenarios 5 and 11 and correlation between X, $Z_{1}$ and $Z_{2}$: Scenarios 6 and 12). Within each scenario the coarsening probabilities, category frequencies and the effect sizes are varied, leading to the 18 sub‐scenarios as described in Section 3.

In Section 3.2.2 the simulation results are illustrated for two specific sub‐scenarios. The simulation results for all 12 scenarios are then discussed in Section 3.2.3.

#### 3.2.1 Infeasible Methods

Due to complete separation between X and C, the two SMC‐FCS methods that directly use C in their imputation model (SMC‐FCS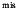 and SMC‐FCS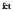) often fail to reach convergence. Depending on the scenario settings, all replications can be subject to this model failure (Table SB1). Because this problem extends to all scenarios and a majority of the sub‐scenarios SMC‐FCS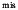 and SMC‐FCS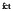 are discarded from further analyses. The issue with the complete separation between X and C is also observed with the two corresponding MICE methods. However, because of implementation of the augmentation method of White et al. [22] in MICE, the models do not fail and coefficients are still estimated in each replication.

#### 3.2.2 Two Sub‐Scenarios

Simulation results are first illustrated with two sub‐scenarios, one from scenario 3 (continuous outcome) and one from scenario 9 (survival outcome). Settings for both sub‐scenarios include a dependence between X and $Z_{1}$ and between X and $Z_{2}$; uniform category frequencies; medium effect sizes and both coarsening and missingness simulated. For both sub‐scenarios, results are shown over all 165 replications.

*Incompatible Classification*

Coarsening is only applied to individuals with a simulated b or c, so imputing an a for such individuals is by definition incorrect. To assess how well the methods prevent these incorrect imputations, the incompatible classification of the coarsened individuals is quantified. Table 2 displays the mean percentage of incompatibly classified individuals, relative to the number of coarsened individuals, for each sub‐scenario. The methods that ignore the coarsening information (MICE and SMC‐FCS) have a high‐level of incompatible classification with percentages around 22%, independent of the simulated outcome. Because C is ignored in these methods, CNAR applies, although because of the correlation between X and $Z_{1}$ and via the outcome some of the coarsening information is retained. For the methods MICE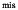 and MICE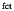, only a few individuals are incompatibly classified, with percentages remaining below 0.1%. In contrast, the methods MICE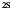, SMC‐FCS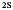 and SMC‐FCS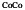 show no incompatible classifications. This is expected as imputations have to be compatible with the coarsening information by construction.

**TABLE 2:** **Incompatible classification of coarsened individuals.** Mean percentages (SD) for the different methods (between brackets the SDs) over all 165 replications.

	Continuous	Survival
MICE	21.99 (1.54)	22.98 (1.51)
MICE 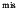	0.07 (0.01)	0.07 (0.02)
MICE 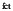	0.02 (0.01)	0.02 (0.01)
MICE 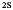	0 (0)	0 (0)
SMC‐FCS	21.82 (1.58)	22.94 (1.48)
SMC‐FCS 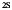	0 (0)	0 (0)
SMC‐FCS 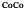	0 (0)	0 (0)

*Coefficients*

**FIGURE 1:**
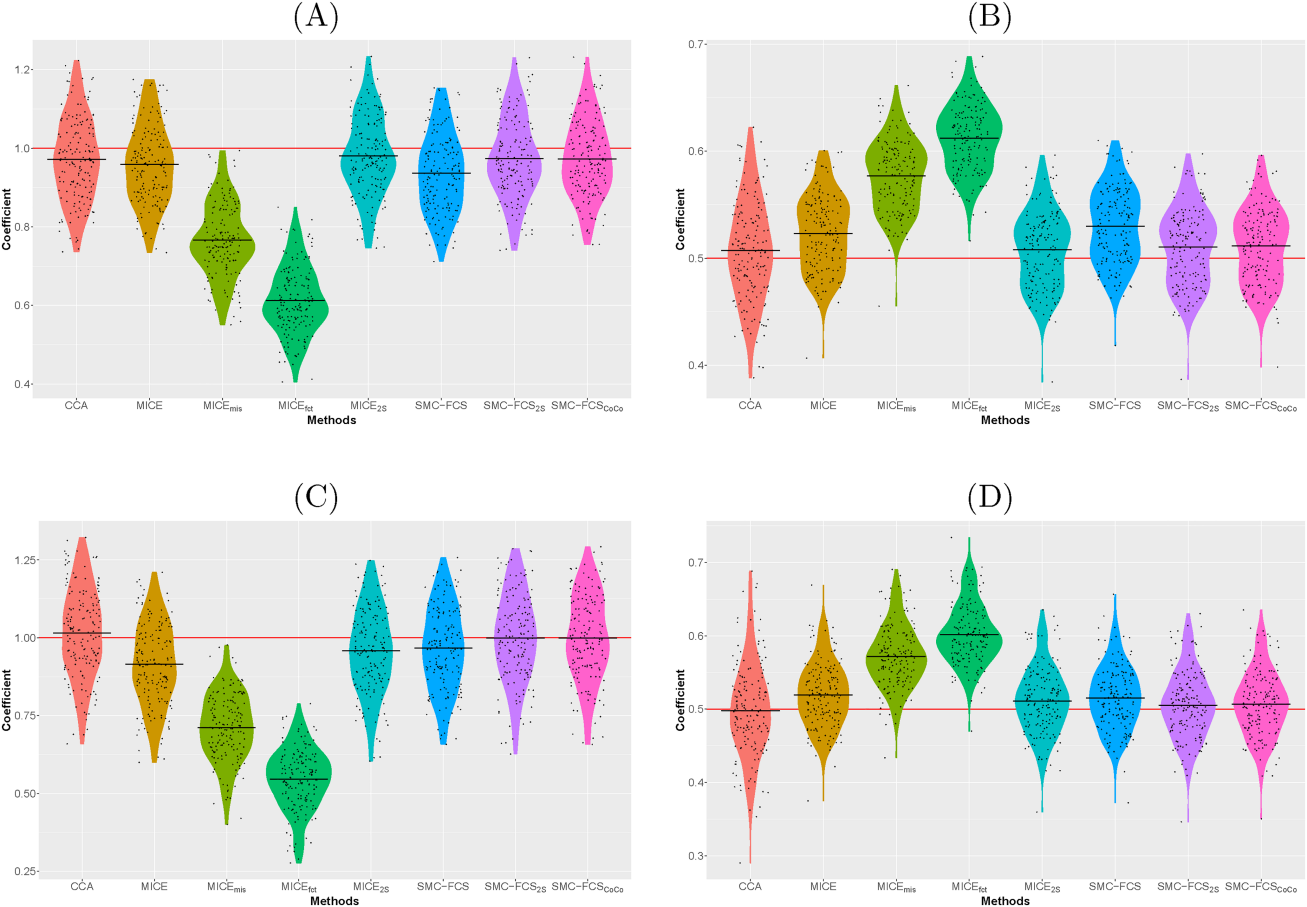
**Distribution of estimated coefficients for the two sub‐scenarios.** Coefficients estimated in all 165 replications are plotted per method. The red horizontal line represents the true value, the horizontal black lines display, per method, the mean coefficient value. (A) {X=c} with continuous outcome, (B) $Z_{1}$ with continuous outcome, (C) {X=c} with survival outcome and (D) $Z_{1}$ with survival outcome.

Coefficients for each covariate level are estimated in each replication. For the two sub‐scenarios, the biases and RMSEs of the coefficients $\beta_{2}$ and $\beta_{3}$ from variables {X=c} and $Z_{1}$ (see Equations (3) and (4)) are shown in Table 3. Figure 1 displays the distributions of the estimates of these coefficients across the replications. For the sub‐scenario with continuous outcome, the methods can be divided into two groups, those that perform well and those that do not. The CCA, MICE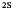, SMC‐FCS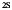 and SMC‐FCS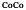 methods perform well, yielding only small bias for all levels. The latter three methods also have comparable low RMSE values, while the RMSEs of the CCA are slightly higher for $\beta_{3}$, the coefficient of $Z_{1}$, probably due to the lower retained sample sizes. Biased estimates are obtained for the other four methods (MICE, MICE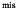, MICE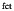 and SMC‐FCS), accompanied with much higher RMSEs.

**TABLE 3:** **Performance two sub‐scenarios.** For the two sub‐scenarios the bias, standard error (SE) of the coefficient, RMSE and 95% confidence interval (CI) coverage is displayed for two regression coefficients: $\beta_{2}$ and $\beta_{3}$ for X=c and $Z_{1}$, respectively. Values in bold are considered good: For the RMSE this entails that the values in bold are at most 10% higher than the minimum value over all methods and for the coverage the values are bold if the estimated coverage lies between 0.925 and 0.975. Performances for the other estimates are given in Tables SB2–B4, for the bias, RMSE and coverage, respectively.

		Continuous				Survival			
		Bias	SE	RMSE	Cov	Bias	SE	RMSE	Cov
X=c	CCA	−0.029	0.105	**0.109**	**0.945**	0.016	0.141	**0.143**	**0.964**
	MICE	−0.042	0.083	**0.108**	0.855	−0.085	0.113	0.156	0.836
	MICE 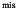	−0.234	0.086	0.251	0.273	−0.289	0.112	0.308	0.285
	MICE 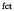	−0.387	0.073	0.396	0.000	−0.454	0.090	0.464	0.000
	MICE 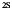	−0.020	0.088	**0.103**	0.903	−0.042	0.121	**0.142**	0.909
	SMC‐FCS	−0.064	0.097	0.118	0.909	−0.033	0.133	**0.137**	**0.952**
	SMC‐FCS 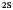	−0.027	0.098	**0.105**	**0.927**	−0.001	0.133	**0.141**	**0.945**
	SMC‐FCS 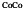	−0.027	0.096	**0.101**	**0.945**	0.000	0.132	**0.138**	**0.952**
$Z_{1}$	CCA	0.007	0.043	0.047	0.921	−0.002	0.067	0.064	**0.945**
	MICE	0.023	0.030	0.042	0.848	0.019	0.042	**0.048**	0.921
	MICE 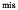	0.077	0.031	0.084	0.339	0.072	0.041	0.083	0.636
	MICE 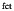	0.112	0.029	0.116	0.030	0.102	0.039	0.110	0.261
	MICE 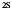	0.008	0.032	**0.037**	**0.927**	0.011	0.043	**0.047**	0.915
	SMC‐FCS	0.030	0.033	0.046	0.818	0.015	0.045	**0.046**	**0.933**
	SMC‐FCS 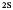	0.010	0.034	**0.037**	**0.945**	0.005	0.045	**0.047**	0.921
	SMC‐FCS 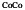	0.011	0.033	**0.037**	**0.933**	0.007	0.045	**0.046**	**0.927**

Similar results are obtained for the sub‐scenario with a survival outcome. The SMC‐FCS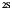 and SMC‐FCS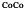 methods remain practically unbiased with low RMSE values and the four other methods still have highest biases and RMSEs. In contrast to the continuous outcome, the MICE methods, including MICE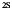, now perform worse than their SMC‐FCS counterparts, which is expected due to the fact that MICE is known to perform worse with non‐linear outcomes. MICE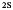 is not unbiased anymore and the MICE method performs worse than SMC‐FCS.

*Coverage*

Table 3 shows the estimated coverage for the two sub‐scenarios. The coverage assesses in how many replications the true coefficient lies within the estimated 95% CI. For the sub‐scenario with a continuous outcome, the same four well‐performing methods (CCA, MICE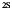, SMC‐FCS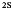 and SMC‐FCS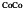) have coverages of about 95%, except for the coverage for {X=c} of MICE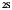 and $Z_{1}$ of CCA. The other four methods perform worse, with the majority of coverages often not close to 95%. Especially the coverages of MICE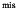 and MICE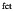 are very low. For the sub‐scenario with a survival outcome, similar coverages are observed, except for the SMC‐FCS method for which all variable levels now also reach correct coverage.

#### 3.2.3 All Scenarios

In this section, simulation results are shown for all 18 sub‐scenarios for each of the 12 scenarios. The two sub‐scenarios discussed above are part of the scenarios 3 and 9, with dependence between X and $Z_{1}$ and between X and $Z_{2}$.

*Incompatible Classification*

The percentage of incompatible classifications for all scenarios is comparable to the percentages observed for the two sub‐scenarios: The methods that ignore C (MICE and SMC‐FCS) have a high percentage of incompatible classification; the two MICE methods with C in their imputation model (MICE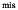 and MICE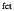) do have some incompatible classifications, although the percentages are very low, while the MICE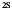, SMC‐FCS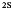 and SMC‐FCS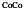 methods have no incompatible classification (Table SB5 and Figure SB1, see Supporting Information). When dependence between X and $Z_{1}$ is introduced, the proportion of incompatible classifications in the MICE and SMC‐FCS methods decreases substantially, where (additional) dependence with $Z_{2}$ has only a minimal effect.

*Coefficients*

**FIGURE 2:**
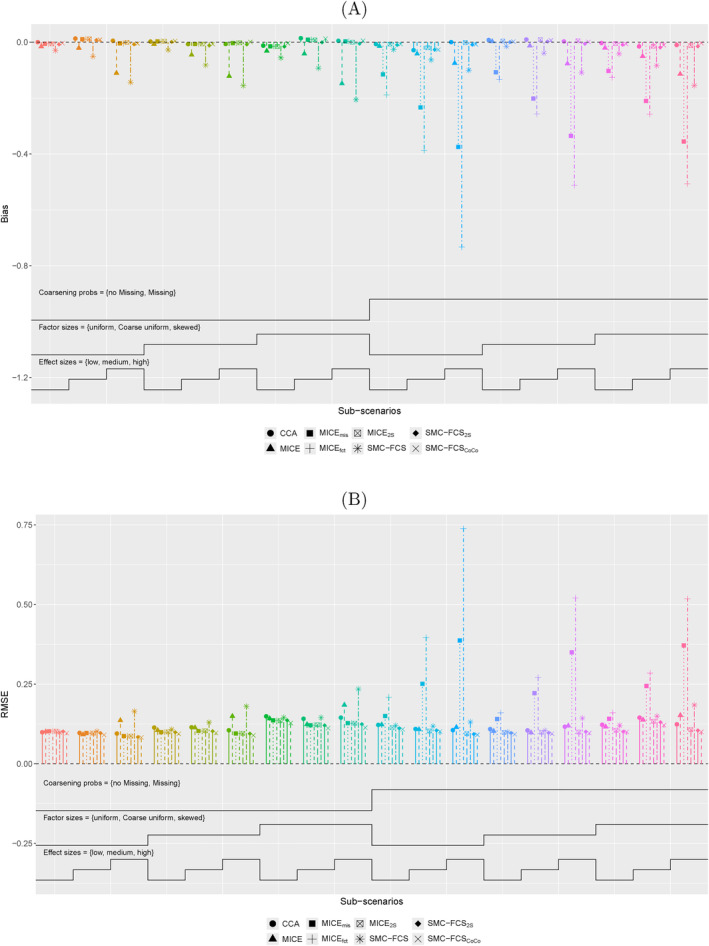
**Nested‐loop plot for coefficients of** {X=c} **with continuous outcome.** The 18 sub‐scenarios of the scenario with dependence between X and $Z_{1}$ and between X and $Z_{2}$ are plotted for (A) bias and (B) RMSE for each of the methods. Each colored set of points depicts a sub‐scenario with a different set of parameters, which are defined by the lines at the bottom of the graph. The sub‐scenarios differ in the probabilities (lower line is only non‐zero coarsening probabilities; upper line is both non‐zero coarsening and missingness probabilities), category frequencies (lowest line is uniform; middle line is b and c uniform and upper line is skewed) and effect sizes (lowest line is low; middle line is medium and upper line is high).

For each method, the bias and RMSE are estimated for all covariate levels. Figure 2 shows the nested‐loop plot for the bias (A) and RMSE (B) of the regression coefficient $\beta_{2}$ of {X=c} for the scenario with dependence between X and $Z_{1}$ and between X and $Z_{2}$, and a normal outcome. When only coarsening is simulated, the error in MICE and SMC‐FCS is much higher than in the other methods, while MICE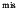 and MICE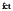 also perform comparably poorly with additional simulated missingness. In contrast, CCA, MICE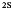, SMC‐FCS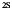 and SMC‐FCS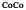 perform comparably well for each sub‐scenario. In general, the impact of varying category frequencies or effect sizes is small, where deviations are only observed for the worse performing methods, with higher errors with higher effect sizes. The biases and RMSEs for $Z_{1}$ are comparable, only CCA now has higher errors in most sub‐scenarios (Figure B2, see Supporting Information).

With other correlation structures, similar patterns are observed. For various sub‐scenarios, the error of SMC‐FCS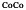 is lower than for all other methods, while its bias remains comparable to the rest (Figure B3, see Supporting Information). The difference with both two‐step methods remains small, however. SMC‐FCS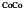 seems to outperform most for {X=c}, as long as there is a dependence simulated between X and $Z_{1}$. This benefit is most profound for the sub‐scenarios where both coarsening and missingness are simulated and act independently of category frequencies or effect size.

**FIGURE 3:**
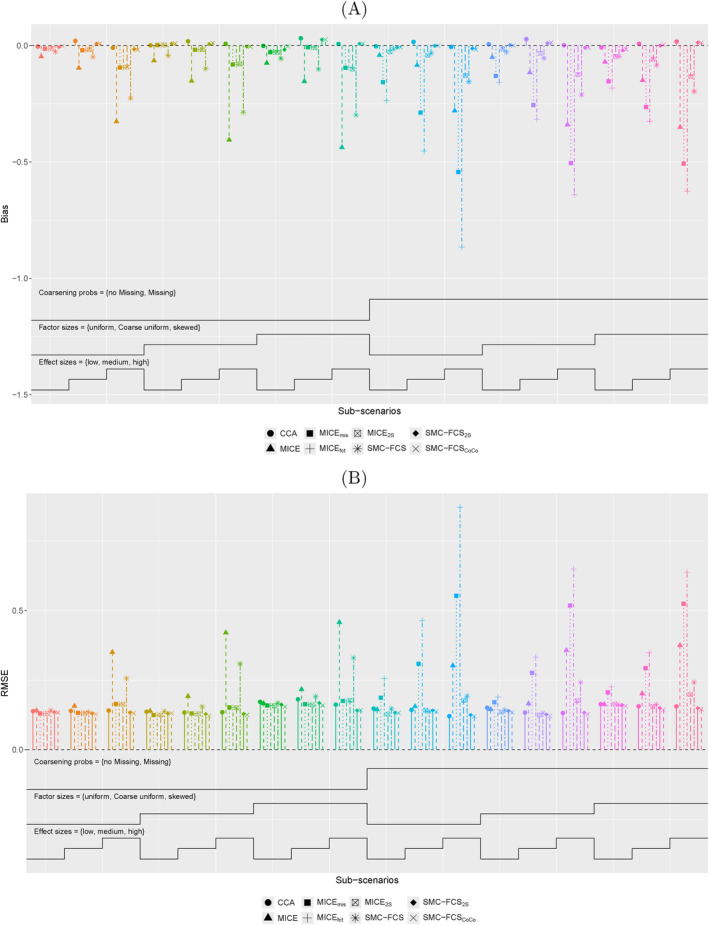
**Nested‐loop plot for coefficients of** {X=c} **with survival outcome.** The 18 sub‐scenarios of the scenario with dependence between X and $Z_{1}$ and between X and $Z_{2}$ are plotted for (A) bias and (B) RMSE. Explanation about the Nested‐loop plots is given in the legend of Figure 2.

With a survival outcome, only the MICE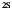, SMC‐FCS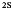 and SMC‐FCS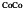 methods show acceptable performance, with substantially higher errors for the other methods (Figures 3 and B4, see Supporting Information). Although, the differences between the MICE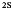 and the two SMC‐FCS approaches (SMC‐FCS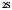 and SMC‐FCS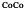) can still become considerable. For most sub‐scenarios of the covariates {X=c} and $Z_{1}$, the two SMC‐FCS approaches have a lower RMSE, which is at least partly because of a lower bias. Interestingly, the error in the MICE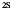 now also increases with higher effect sizes at various sub‐scenarios, where the SMC‐FCS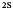 and SMC‐FCS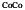 methods remain insensitive for varying effect sizes. With other correlation structures, the two SMC‐FCS methods still perform best overall (Figure B5, see Supporting Information).

*Coverage*

Table SB6 shows the mean coverage across all sub‐scenarios for the scenarios with dependence between X and $Z_{1}$ and between X and $Z_{2}$ and a continuous outcome. Similar as before, MICE, MICE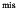, MICE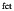 and SMC‐FCS have low coverage for at least some of the variable levels. The variation in the sub‐scenarios for these methods is large, where coverage is especially low when both coarsening and missingness are simulated and when effect sizes are higher. The variation for the other methods, CCA, MICE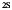, SMC‐FCS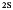 and SMC‐FCS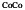, is low, with good coverages for all. Only for {X=c} for MICE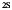 coverage is below 0.9.

For the sub‐scenarios with a survival outcome similar coverages are observed, with a clear separation between the well and badly performing methods (Table SB7). Only the MICE methods perform slightly worse, with the coverage for {X=c} of MICE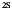 dropping further.

## 4 Illustration

The illustration consists of a collection of four cohorts (PORTEC‐1, PORTEC‐2, PORTEC‐3 and MST), all investigating the best adjuvant treatment strategy for patients suffering from endometrial carcinoma [13, 14, 15, 16]. A total of 2071 patients were followed over time, with a median follow‐up of 10.0 years. Important clinical risk factors for endometrial carcinoma include patient age, disease stage, histological type and LVSI. The latter is subject to coarsening, where for 410 patients it is only known that lymphovascular space invasion is present, mainly from the PORTEC‐3 cohort (Table 4). For 15 individuals from the MST cohort, the LVSI observations are missing, together with their histological type.

**TABLE 4:** **Observations of LVSI.** The number of patients with the different LVSI observations per cohort. Individuals that are scored as present are coarsened. Their true observation is either focally present or substantially present, but certainly not absent.

	PORTEC‐1	PORTEC‐2	PORTEC‐3	MST
Absent	641	344	271	152
Focally present	36	53	0	50
Substantially present	26	20	0	53
Present	11	10	389	0
Missing	0	0	0	15

Each method described in Sections 2.1 and 2.2 was applied to the PORTEC and MST data. For each method, a Cox model was run with the time to recurrence free survival (RFS) as the event of interest. A total of 832 patients experienced either death or recurrence during follow‐up, the other patients were censored at last follow‐up. The clinical covariates patient age, disease stage, histological type, and LVSI and the RFS time and status indicator were used for the imputation and analysis model. For the MICE methods, the RFS time was excluded from the imputation method and replaced by the Nelson‐Aalen estimate of the cumulative marginal hazard [21]. For the methods that use C as predictor in the imputation model, C was also added to the imputation model of LVSI and histological type, and vice versa. For the two‐step methods, histological type was only imputed in the second step, since it only occurred in combination with missing (and not coarsened) LVSI observations.

As with the simulation study, the SMC‐FCS methods with C in the imputation model (SMC‐FCS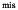 and SMC‐FCS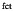) did not converge due to complete separation between X and C. Therefore, the analyses were only run for the other eight methods. The methods without coarsening information have high‐percentages of incompatible classification (in all replications >80%), while in contrast, all other methods impute the coarsened individuals always into one of the two compatible levels (Table SB8).

**FIGURE 4:**
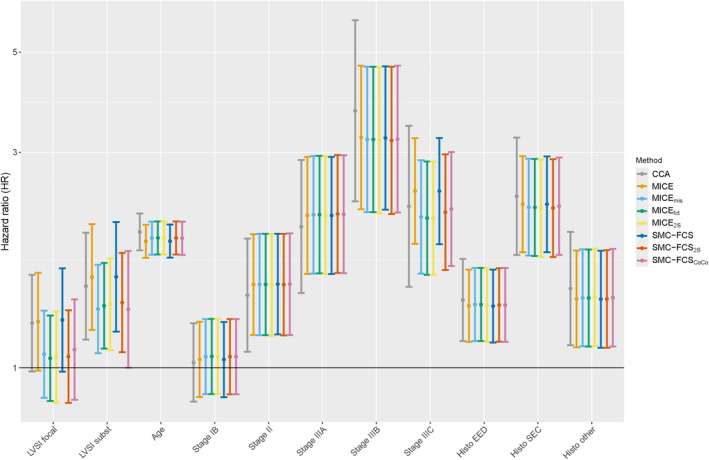
**Real data forest plot**. Hazard ratios (95% CI) are shown for each covariate level for the eight different methods.

Based on the coefficients for LVSI, the methods can be roughly divided into two groups (Figure 4 and Table SB8, see Supporting Information). The methods CCA, MICE and SMC‐FCS have comparable estimates that are higher than those obtained by the other methods. The differences between the other five methods are small for the focally LVSI and slightly bigger for substantial LVSI. For the latter, the SMC‐FCS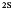 and SMC‐FCS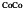 methods perform comparably and are most divergent with the MICE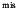 and MICE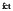 methods. The MICE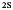 method has an estimated coefficient between these two groups of methods. The coefficients of the other covariates are similar for all methods, except for the CCA for which also the standard errors are inflated compared to the rest and the coefficients for disease stage IIIC, which shows a similar method division as with LVSI.

## 5 Discussion

In this study, we developed a new method for dealing with coarsened and missing data in multiple imputation. This method was compared to a number of ad hoc approaches which could be considered as alternatives in practice. Coarsening is a broad term for various forms of partly observed data. It includes censoring, grouping and rounding as special cases, each coming with its implications and underlying assumptions. Here, we have used the term coarsening to mean that for some individuals a subset of the full sample space is observed that includes the true underlying observation, instead of the true observation itself. To our knowledge, no clear‐cut method has been described to impute coarsened observations in a regression context. We therefore developed a principled approach to this problem, consisting of an adaptation of SMC‐FCS, and suggested a number of plausible ad hoc approaches, possibly already in use by statisticians. We compared these in a simulation study. These ad hoc methods cover a variety of different approaches, although different choices could have been made.

Based on the motivating study, we chose to investigate a data problem with a categorical variable with three categories, where for a subset of individuals coarsened observations {b,c} are observed instead of either b or c. Extensions to this simple data problem are simple to imagine, with coarsening being present also for other category combinations, such as {a,b} or {a,c}. Most presented methods are cumbersome to extend to such a situation, since the coarsening indicator C has to be constructed in a different way. The proposed SMC‐FCS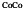 method has no difficulty with accommodating such more complex coarsening structures, is easy to use and is implemented in the {smcfcs} package in R (see Appendix A).

The simulation study shows that the methods that ensure that coarsened observations are imputed with a value that is compatible with the coarsened information, that is, MICE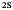, SMC‐FCS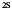 and SMC‐FCS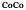, perform consistently better, in terms of a lower bias and RMSE and better coverage, than the other methods that ignore the coarsening information, or handle it in a more naïve way. Both two‐step methods (MICE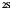 and SMC‐FCS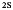) impute values based only on the observations that are compatible with the coarsening information, while the SMC‐FCS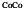 method imputes values based on Equation (2), thus preventing incompatible imputations. Additionally, the two SMC‐FCS methods also perform better than MICE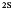 for certain scenarios, especially when survival outcomes are simulated.

Results of methods in the real data analysis showed heterogeneity. The methods can be divided into two groups: The CCA and the two methods that ignore the coarsening information versus the methods that (in)directly implement the coarsening information in the imputations. Because in the real data there is a majority of observations with absent LVSI (a), the methods that impute X in a naïve way (MICE and SMC‐FCS) have a very high‐percentage of incompatible classifications (>80%). These results are therefore likely to be invalid. For the other group of methods, both SMC‐FCS methods show very similar results to MICE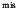 and MICE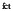 for the effect of focal LVSI, and comparable effect size for the substantial LVSI level. For the latter somewhat higher effects were observed for the SMC‐FCS and MICE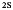 methods.

Although in the real data analysis the MICE methods MICE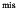 and MICE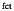 perform comparably to the other methods, the simulation study shows that they can lead to high‐bias and RMSE and unfavorable coverage under several scenarios. Especially in the scenarios where both coarsening and missingness are simulated, the two methods can perform poorly. However, in the MST cohort, there were only 15 missing LVSI observations (<1%), which were probably not influential enough to cause any problems.

With the MICE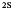, SMC‐FCS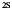 and SMC‐FCS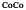 methods, estimates were performing well in general. However, a potential practical problem with the two‐step methods is their lack of generality. Both methods impute based on a subset of the observations, which can become difficult to manage with multiple levels of coarsening or problematic when the sample size of the subset becomes low. Additionally, there is the difficulty of how to impute the missing observations in other covariates. In contrast, since for the SMC‐FCS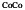 method only the imputation probabilities are altered, it is simple to extend without adding much computational complexity and it imputes all variables in a single step. It would probably also be possible to adjust the MICE algorithm to work in a similar way. The *exclude* argument in {mice} already allows for excluding certain categories from the imputation procedure, although this exclusion has to be the same for all cases. This feature can directly be applied to a coarsening problem with only one type of coarsening and no completely missing data or implemented in a procedure which is in essence the same as the two‐step methods presented earlier. It is however not as straightforward to be applied on the same scale as SMC‐FCS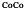. We only implemented it for the SMC‐FCS algorithm because the SMC‐FCS algorithm performs better for non‐linear relations [3]. This superior performance of the SMC‐FCS was also observed here, implying it extends to scenarios with coarsened data.

A difficulty with the SMC‐FCS algorithm is dealing with perfect separation between covariates with more than two categories. SMC‐FCS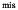 and SMC‐FCS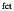 could not be evaluated because many imputation models did not converge due to the perfect separation between X and C, leading to improper imputations. In MICE, the data augmentation of White et al. [22] is implemented, which concatenates pseudo‐observations with a small weight to the data, thereby avoiding infinite estimates [2]. This approach assumes that although the observation is not observed, it could still occur in the population. However, it is known that this is not the case for coarsened data, because when we know LVSI is present, it can never be also absent. Related issues with how to deal with perfect separation are discussed previously [22]. Because this was out of the scope of this article, we did not pursue this.

The real data example is based on the PORTEC and MST studies, which aim to determine the best adjuvant treatment strategy for patients with endometrial carcinoma [13, 14, 15, 16]. Results presented here are in line with what was found previously, with the effect of substantive LVSI more profound than focal LVSI. However, focal LVSI was not significantly different from absent, which has been observed earlier [12]. It has to be kept in mind that the analysis presented here is undertaken with the aim of evaluating the different methods to deal with coarsening, not to estimate a causal relation between predictors and outcome or to optimize prediction accuracy. Therefore, only a limited set of covariates, which have an assumed correlation with the coarsened variable LVSI, has been included and no other modeling strategies were investigated. Interpretation of the results should thus be taken with care.

Although examples of coarsening are easy to imagine, coarsening is rarely described in literature. In contrast, the completely missing framework is extensive, with many theoretical and practical implementations. Both data processes have similar assumptions, where especially CAR and MAR are standard assumptions in practice. Under CAR, it is assumed that each value within an observed coarsened subset has the same probability of becoming coarsened, given the other observed data. Like the MAR assumption, also the CAR assumption is untestable. But even correct specification of the imputation model can prove to be insufficient. In our previous studies investigating coarsening in the genotyping process of the *KIR* gene region, the imputation model had to operate in a high‐dimensional setting, due to the vast number of genotype options, and was unable to correctly estimate effect sizes [8].

This omics example points out that the problem of coarsening is much broader than clinical covariates. The continued refinement of measurement technology creates data sets that can be viewed as being coarsened relative to each other. Examples include genotyping by SNP arrays versus sequencing [23] or increasing sequencing depth in gene expression data [24].

In summary, we have presented an extension to the SMC‐FCS algorithm that handles a coarsened data problem appropriately. For categorical covariates our extension is simple to apply as it requires limited additional computational cost and is straightforward to extend to multiple coarsening settings.

## Conflicts of Interest

The authors declare no conflicts of interest.

## Supporting information


**Figures S1–S5, Supporting Information**.

## Data Availability

The data that support the findings of this study are available on request from the corresponding author. The data are not publicly available due to privacy or ethical restrictions.

## Appendix A A: SMC‐FCS Implementation

For the implementation of the SMC‐FCS method in the {smcfcs} package in R, an additional argument was added: *Restrictions*. This argument requires a list of vectors of strings that indicates whether the variable is a coarsened variable. For variables for which no coarsening is observed, this needs to be indicated with an empty string. Each string of a coarsened variable has to contain three elements in the form of $X_{\text{obs}}=\{b,c\}∼b+c$:

- Variable name ($X_{\text{obs}}$): The variable in the dataset that contains the coarsening information.
- Value ({b,c}): The individuals for which additional coarsening information is available are the set of individuals who, for the specified variable ($X_{\text{obs}}$) have an observation that matches the value.
- Options (b+c): The options that the observations of the coarsened variable can be.

It is possible to omit the third element (the “Options” element), then the possible options are extracted from the name of the value. In this case, the value name consists of b and c, so that are considered to be the only possible options.

For the analysis of the dataset as in Table 1, we would have a dataset with 5 columns in the following order: $X_{\text{obs}}$, X, $Z_{1}$, $Z_{2}$ and Y. Coarsening is observed in variable X (the second column), where the coarsening information is stored in variable $X_{\text{obs}}$ (the observed information). To make that clear to the algorithm, the arguments would look like

Because a string is specified for thes second element of *restrictions* (which corresponds to the X column), the algorithm will detect additional information for X. This additional information comes from the column $X_{\text{obs}}$, for the observations that match value {b,c} where the X values for those observations should either be b or c. The…in the smcfcs function indicate other required parameters. For the completely missing observations, nothing needs to be specified. Since their value of C is not {b,c}, no alterations in the code are made.

*Multiple Ways of Coarsening*

When multiple variants of coarsening are observed for a single variable, this can be indicated with multiple strings in the list element. For example, when in X there would also be coarsening between {X=a} and {X=b} ({a,b}). This analysis can then be performed as
