# Supplementary figures and images for: Analyzing Coarsened and Missing Data by Imputation Methods
Source: Stat Med. 2025 Mar 5;44(6):e70032. doi: 10.1002/sim.70032 (PMC11881681; doi:10.1002/sim.70032)

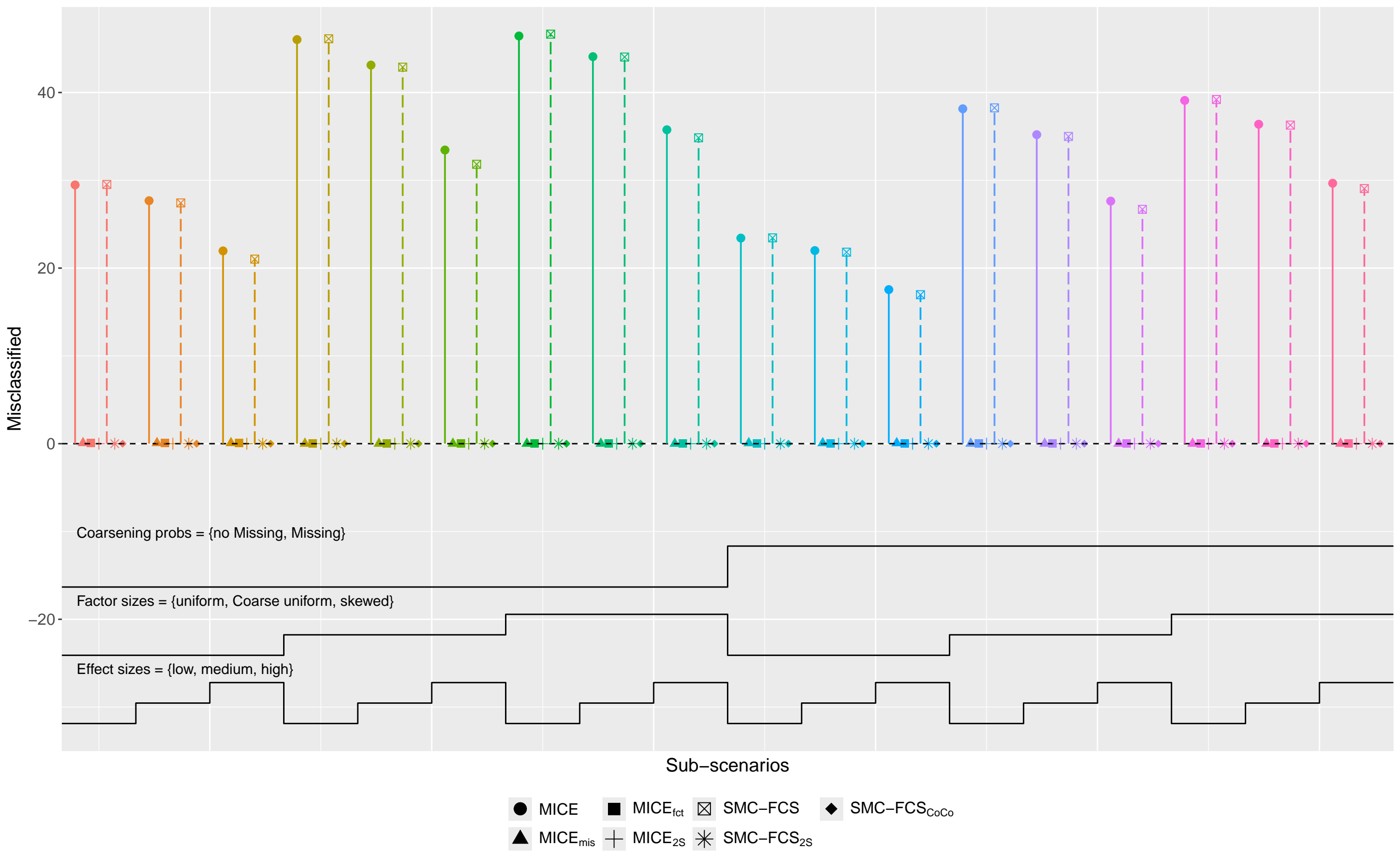

Supplement: Supplementary file 1 — Figures S1–S5, Supporting Information. [file SIM-44-0-s001.zip › Figure_S1A_norm_misclas.pdf]

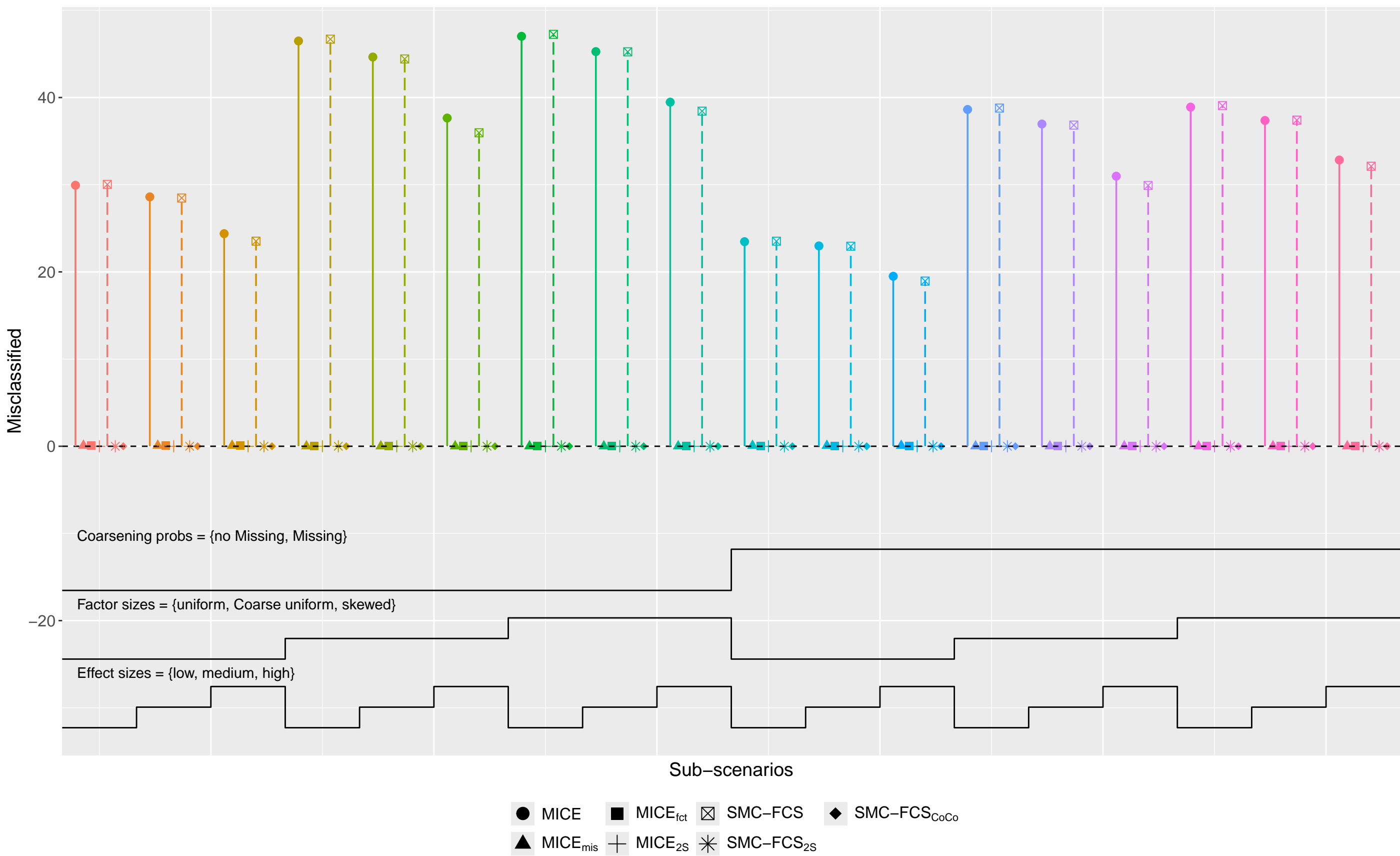

Supplement: Supplementary file 1 — Figures S1–S5, Supporting Information. [file SIM-44-0-s001.zip › Figure_S1B_surv_misclas.pdf]

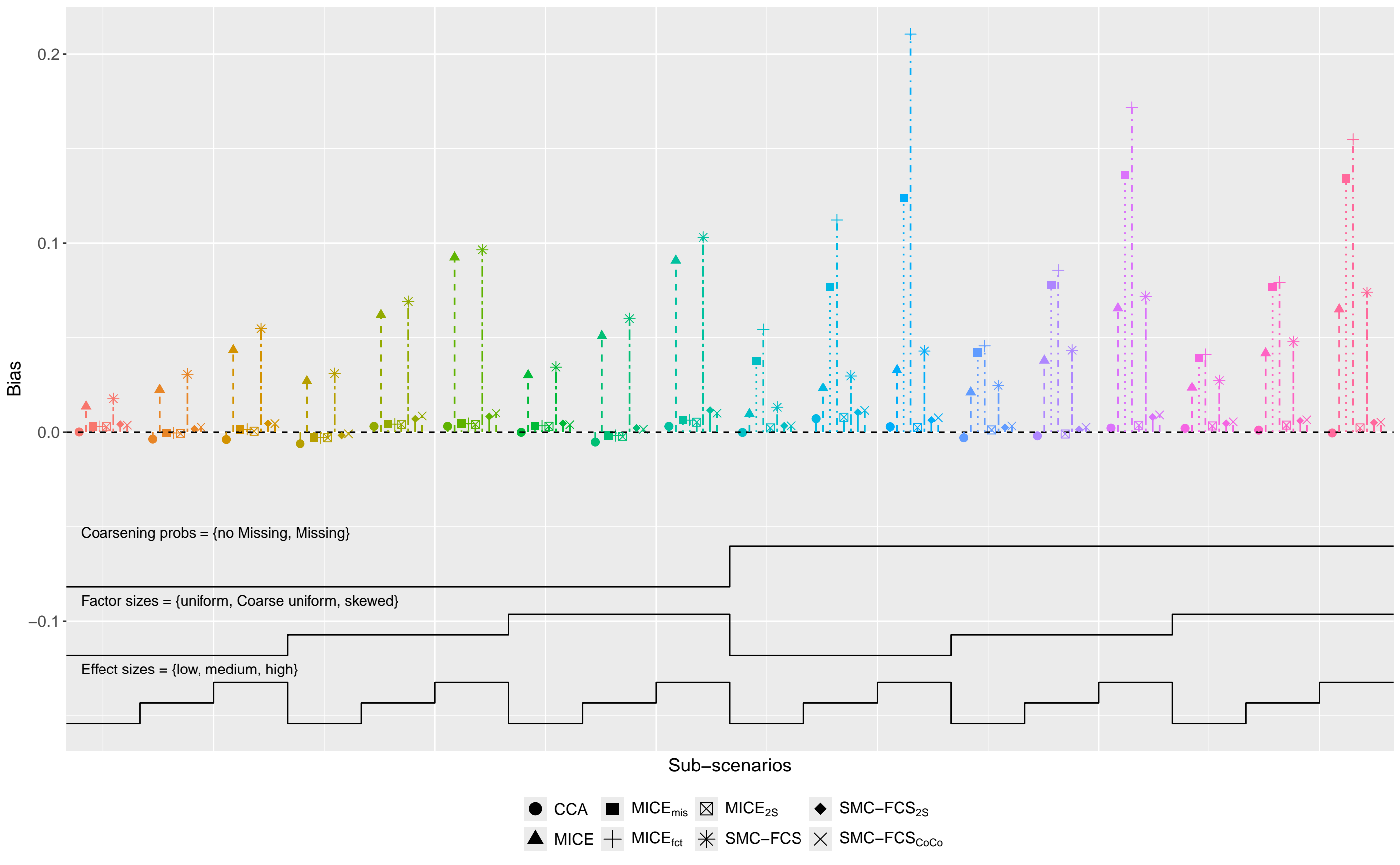

Supplement: Supplementary file 1 — Figures S1–S5, Supporting Information. [file SIM-44-0-s001.zip › Figure_S2A_norm_Bias_Z1.pdf]

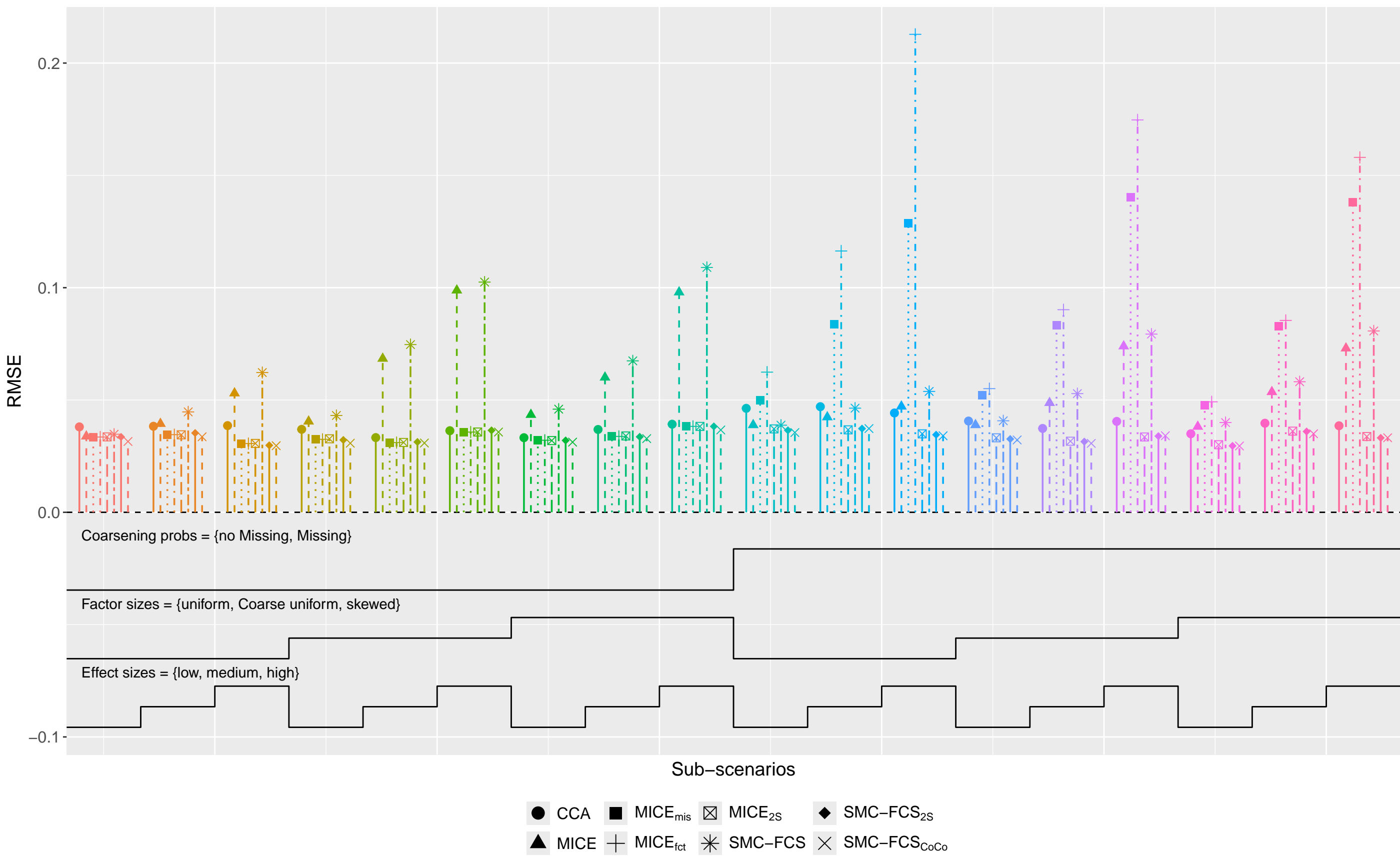

Supplement: Supplementary file 1 — Figures S1–S5, Supporting Information. [file SIM-44-0-s001.zip › Figure_S2B_norm_RMSE_Z1.pdf]

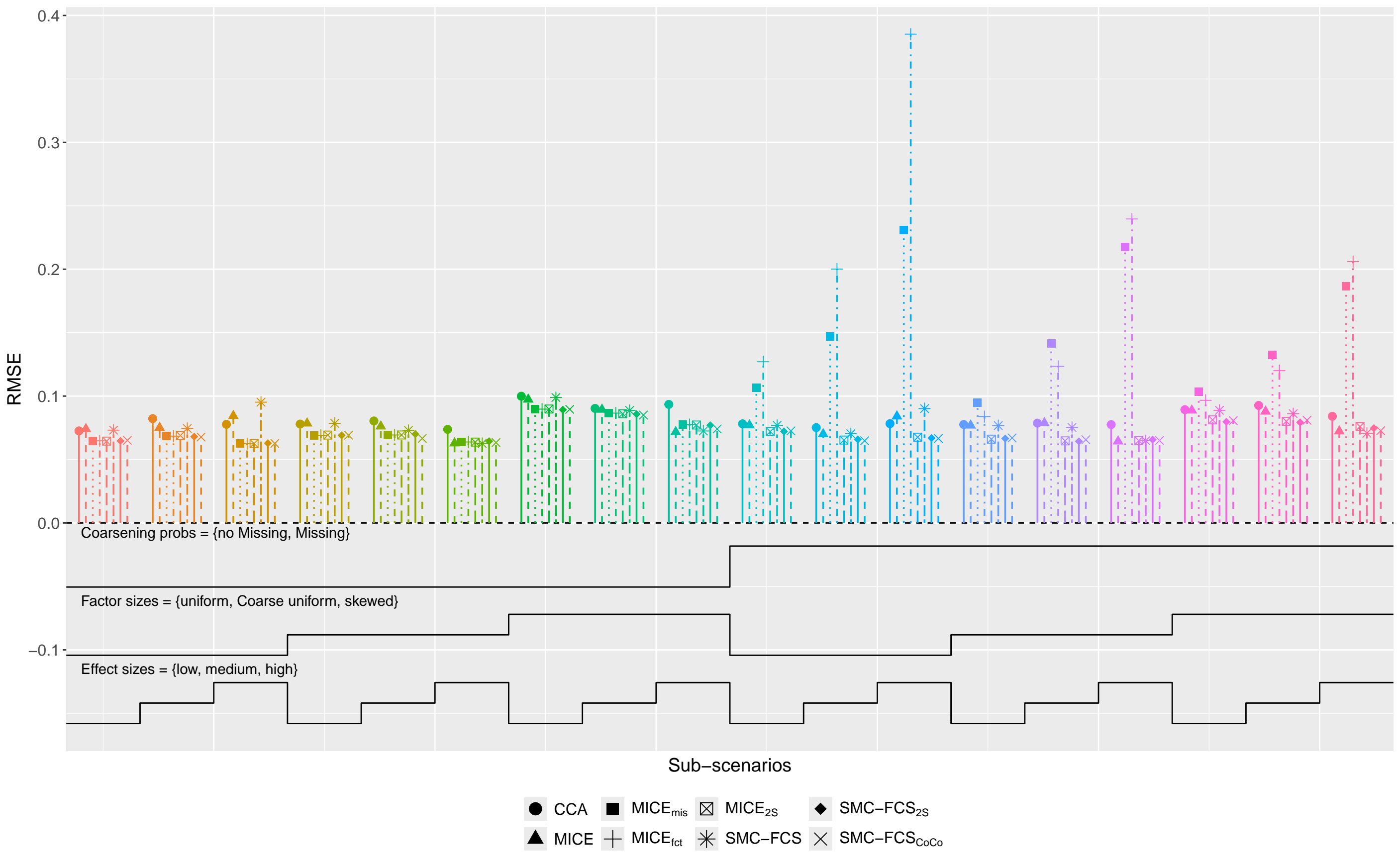

Supplement: Supplementary file 1 — Figures S1–S5, Supporting Information. [file SIM-44-0-s001.zip › Figure_S3A_norm_RMSE_Xc.pdf]

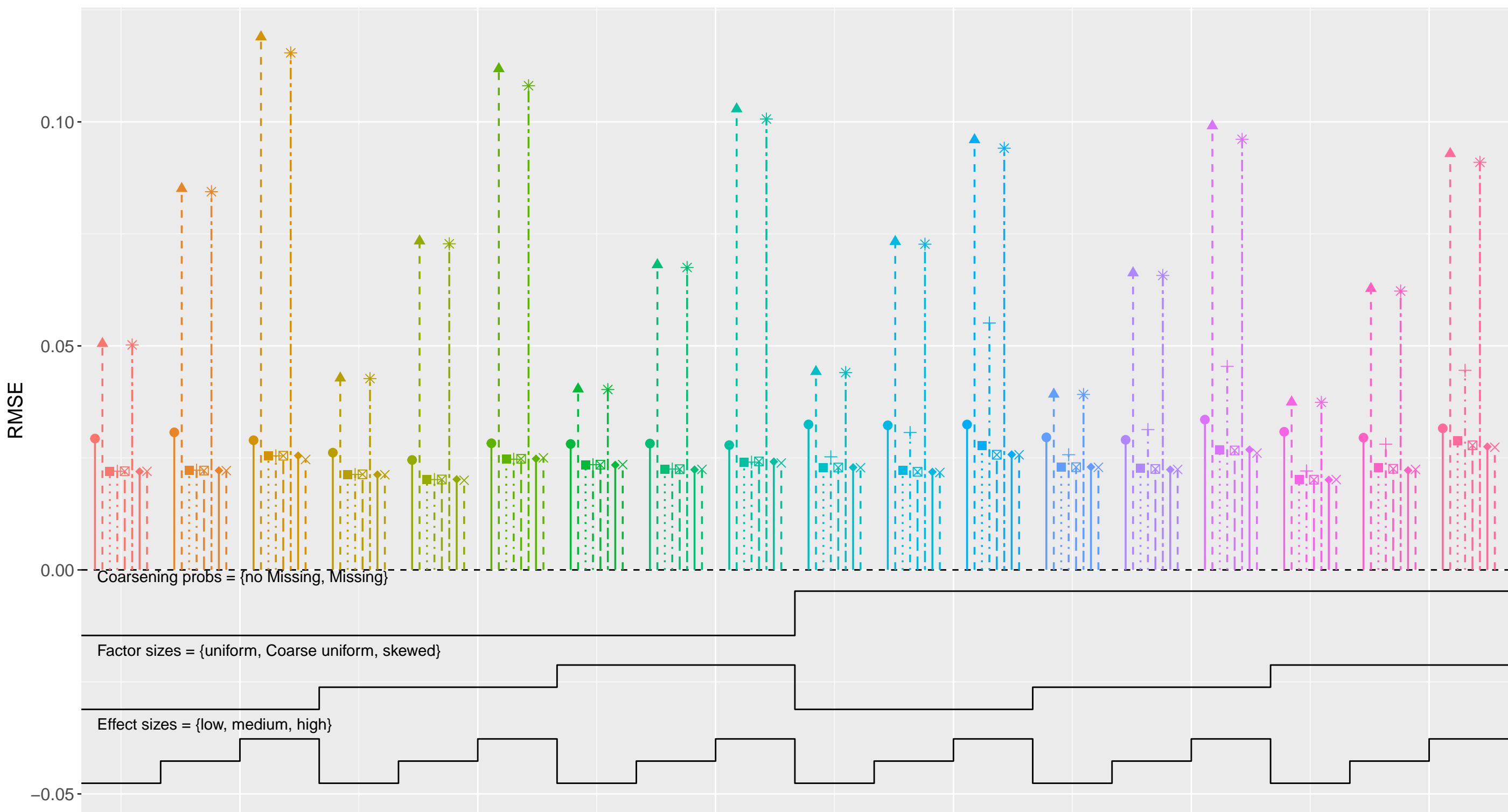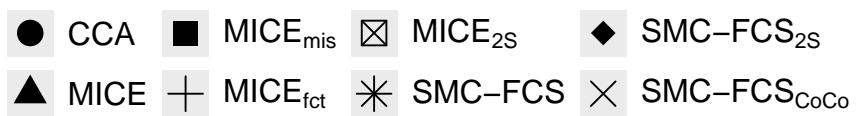

Supplement: Supplementary file 1 — Figures S1–S5, Supporting Information. [file SIM-44-0-s001.zip › Figure_S3B_norm_RMSE_Z1.pdf]

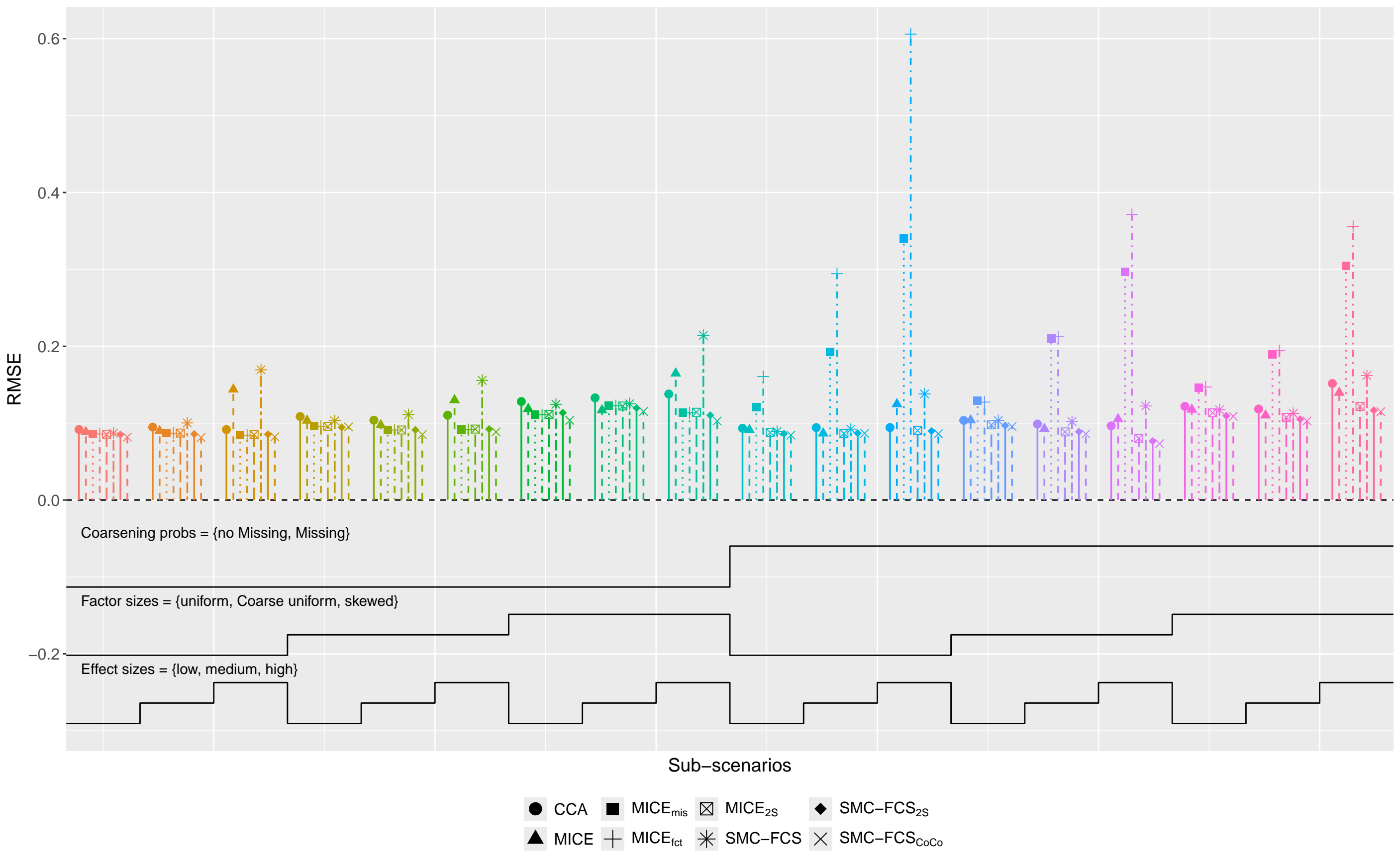

Supplement: Supplementary file 1 — Figures S1–S5, Supporting Information. [file SIM-44-0-s001.zip › Figure_S3C_norm_RMSE_Xc.pdf]

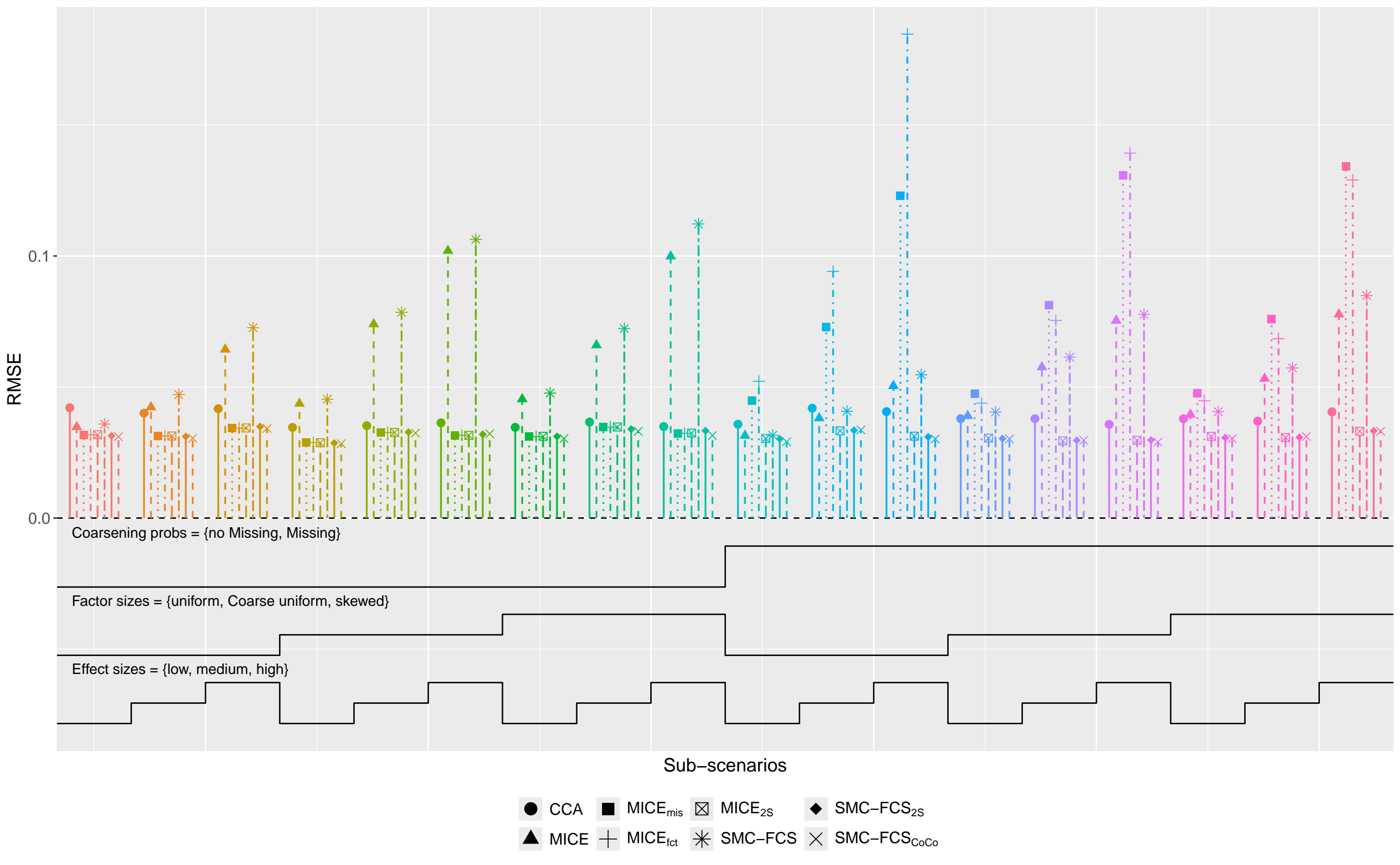

Supplement: Supplementary file 1 — Figures S1–S5, Supporting Information. [file SIM-44-0-s001.zip › Figure_S3D_norm_RMSE_Z1.pdf]

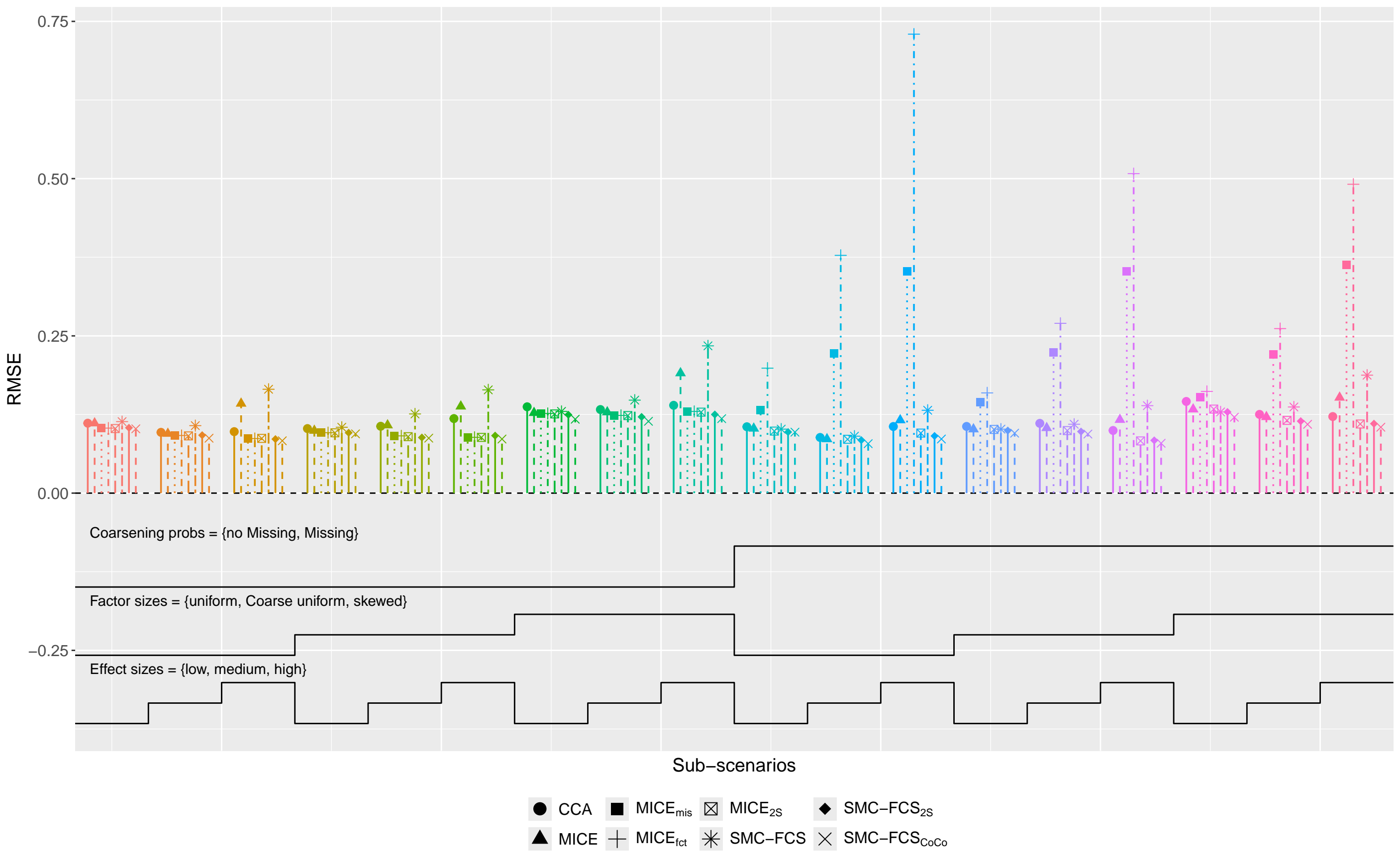

Supplement: Supplementary file 1 — Figures S1–S5, Supporting Information. [file SIM-44-0-s001.zip › Figure_S3E_norm_RMSE_Xc.pdf]

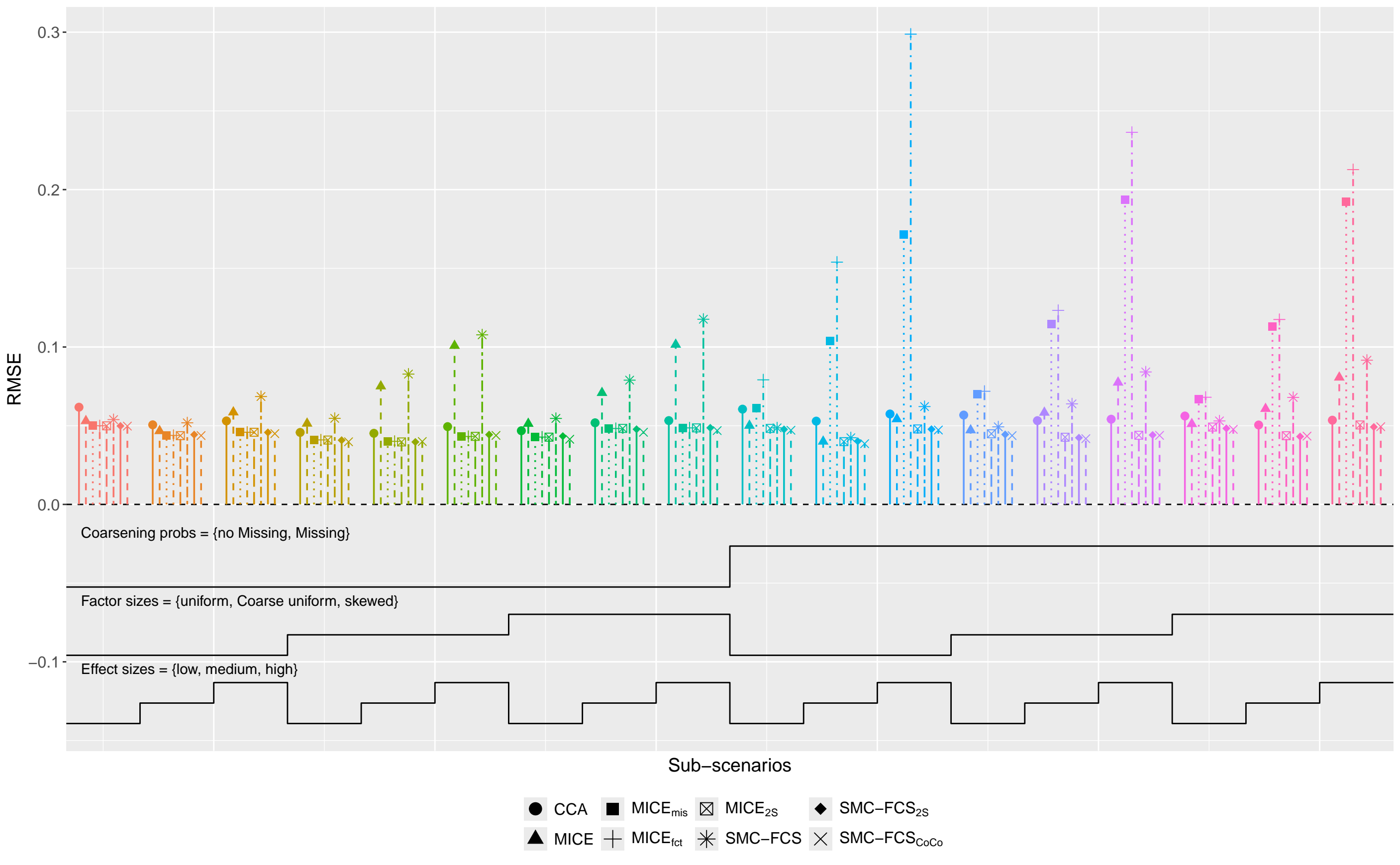

Supplement: Supplementary file 1 — Figures S1–S5, Supporting Information. [file SIM-44-0-s001.zip › Figure_S3F_norm_RMSE_Z1.pdf]

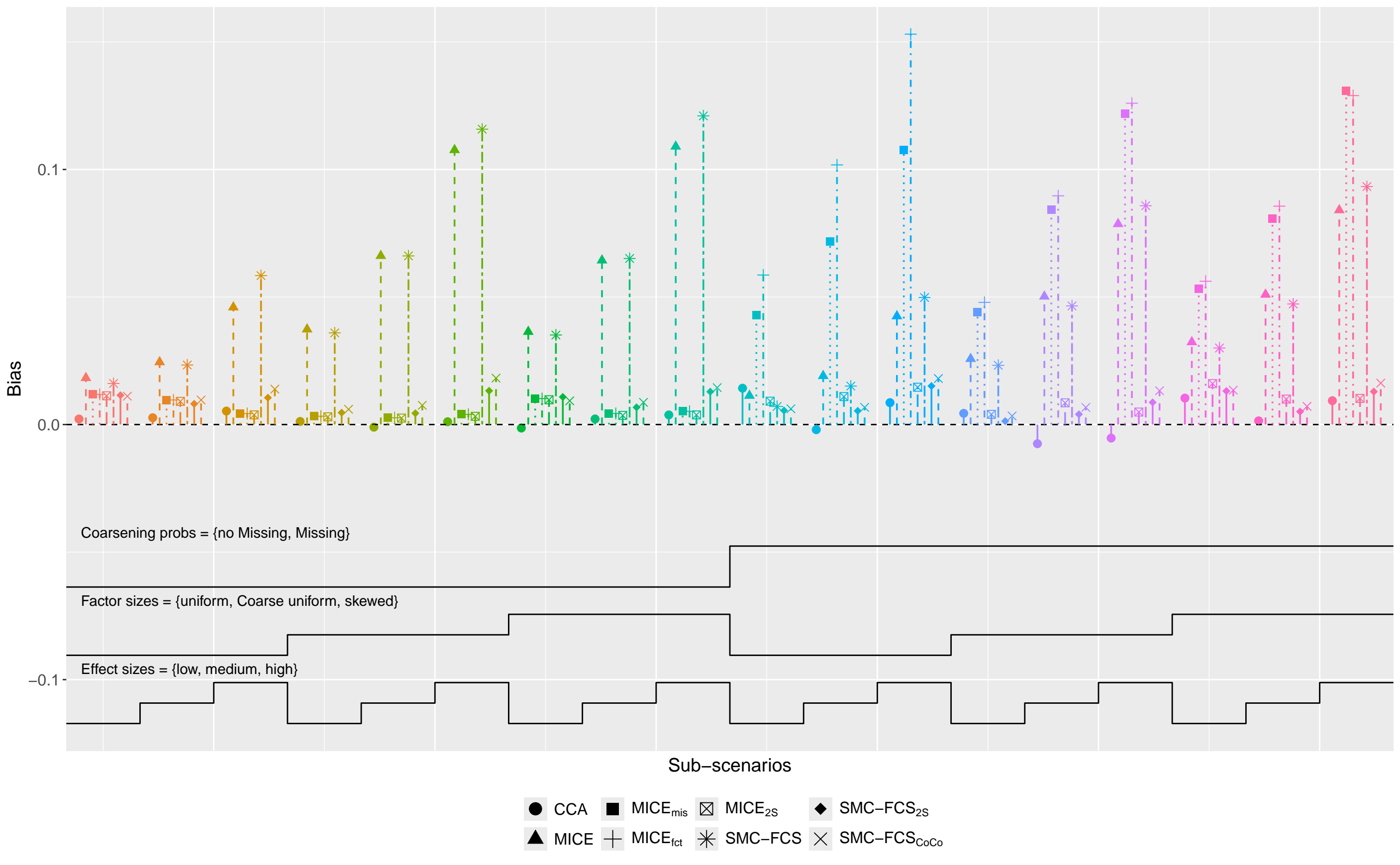

Supplement: Supplementary file 1 — Figures S1–S5, Supporting Information. [file SIM-44-0-s001.zip › Figure_S4A_surv_Bias_Xc.pdf]

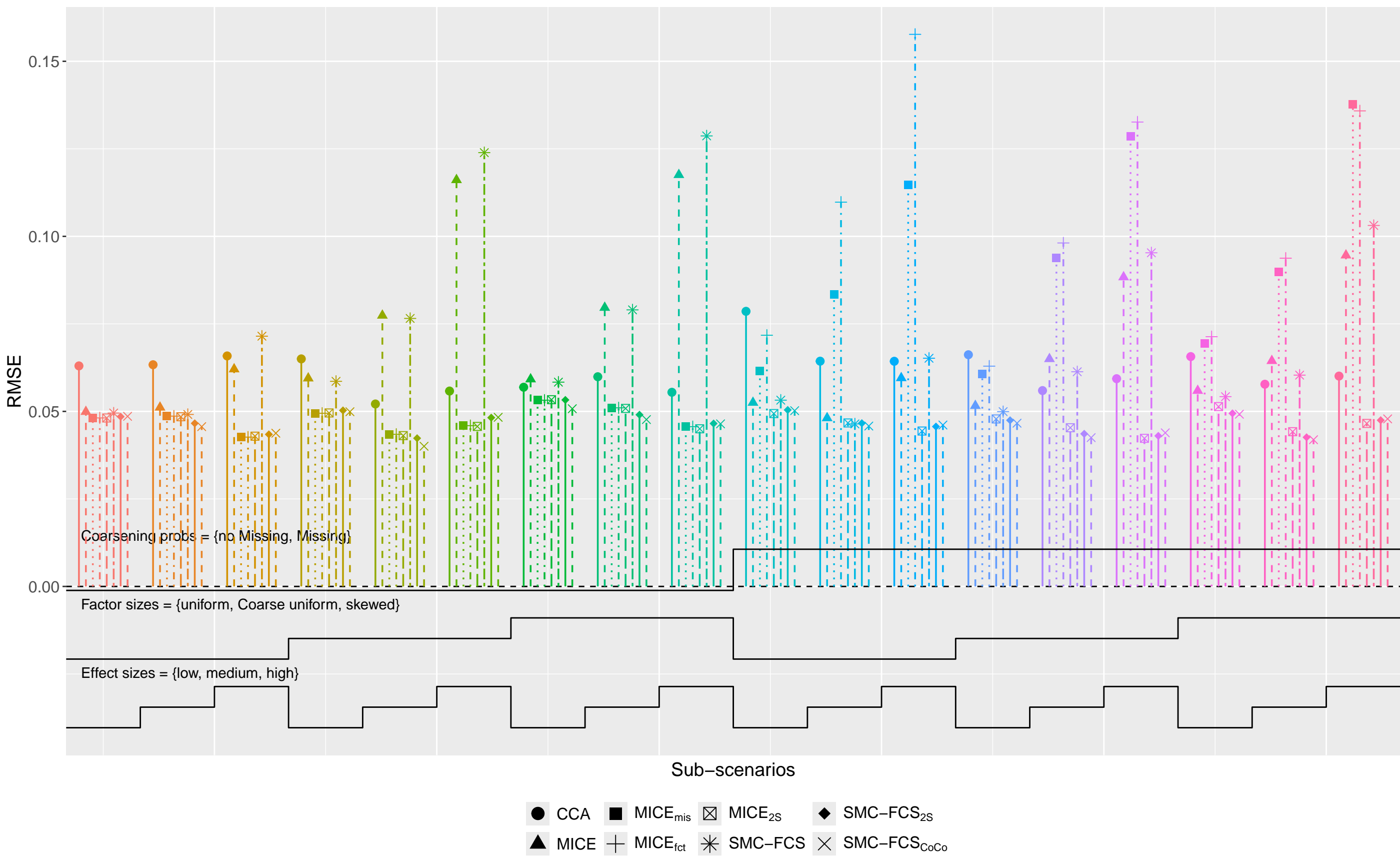

Supplement: Supplementary file 1 — Figures S1–S5, Supporting Information. [file SIM-44-0-s001.zip › Figure_S4B_surv_RMSE_Xc.pdf]

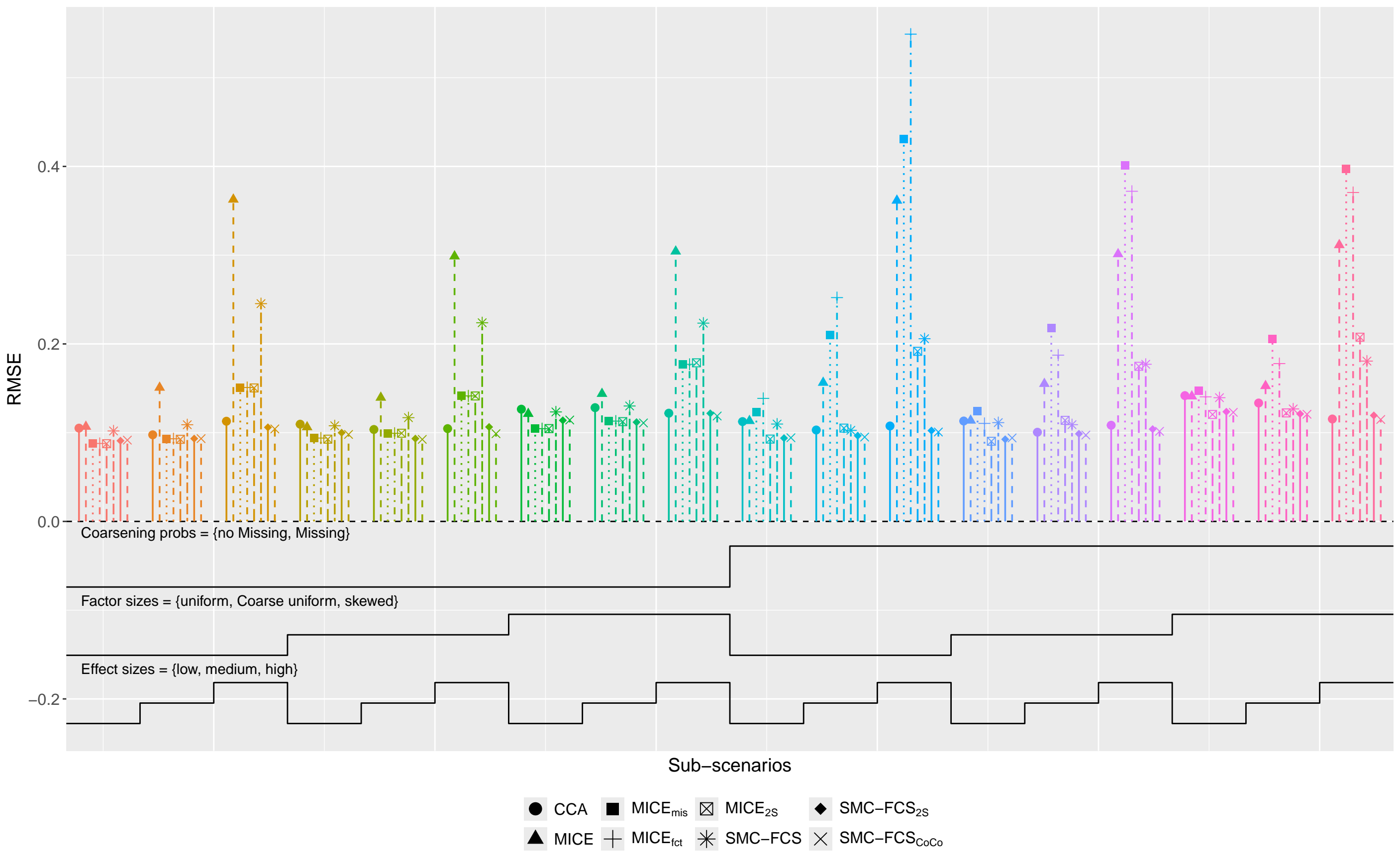

Supplement: Supplementary file 1 — Figures S1–S5, Supporting Information. [file SIM-44-0-s001.zip › Figure_S5A_surv_RMSE_Xc.pdf]

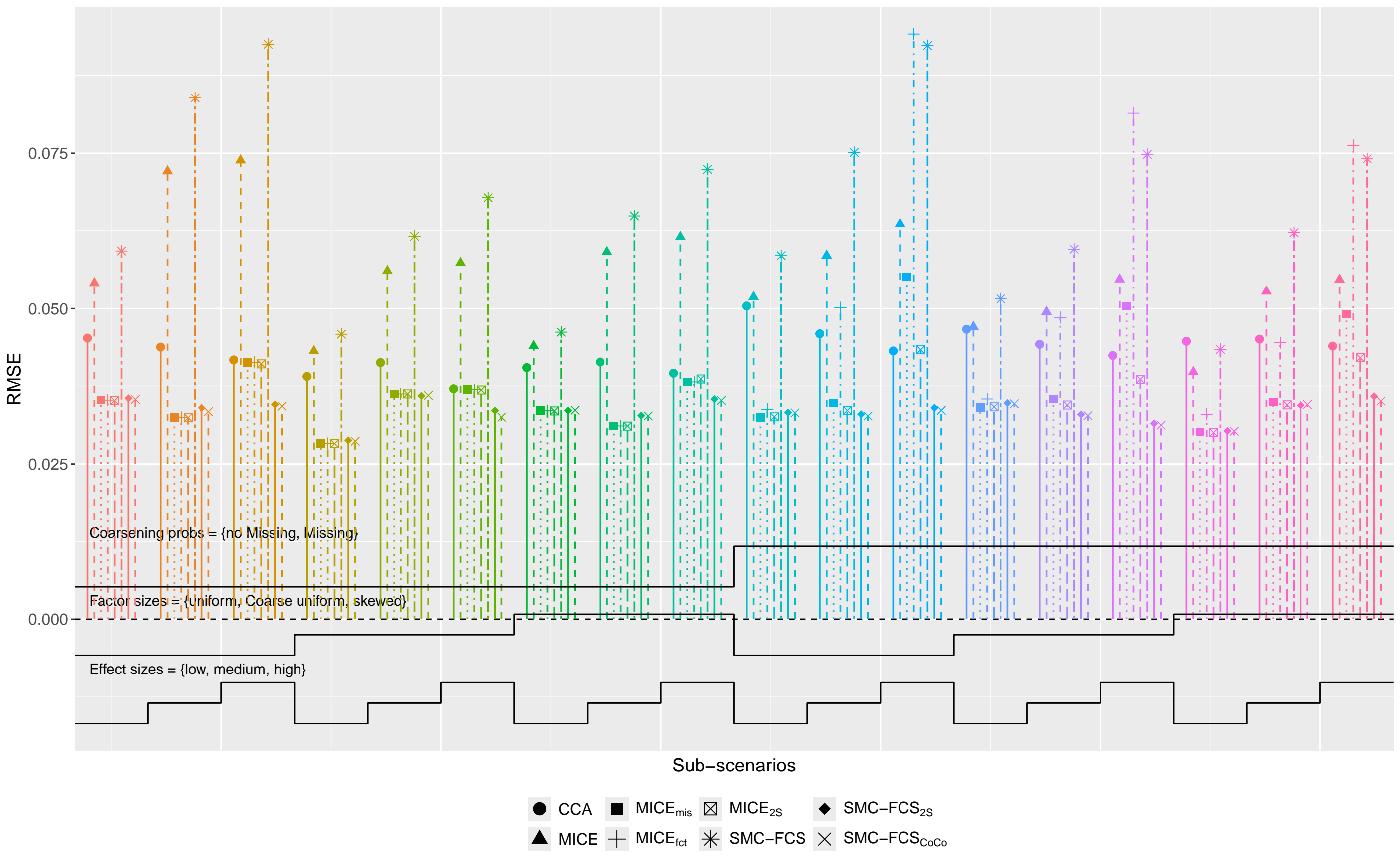

Supplement: Supplementary file 1 — Figures S1–S5, Supporting Information. [file SIM-44-0-s001.zip › Figure_S5B_surv_RMSE_Z1.pdf]

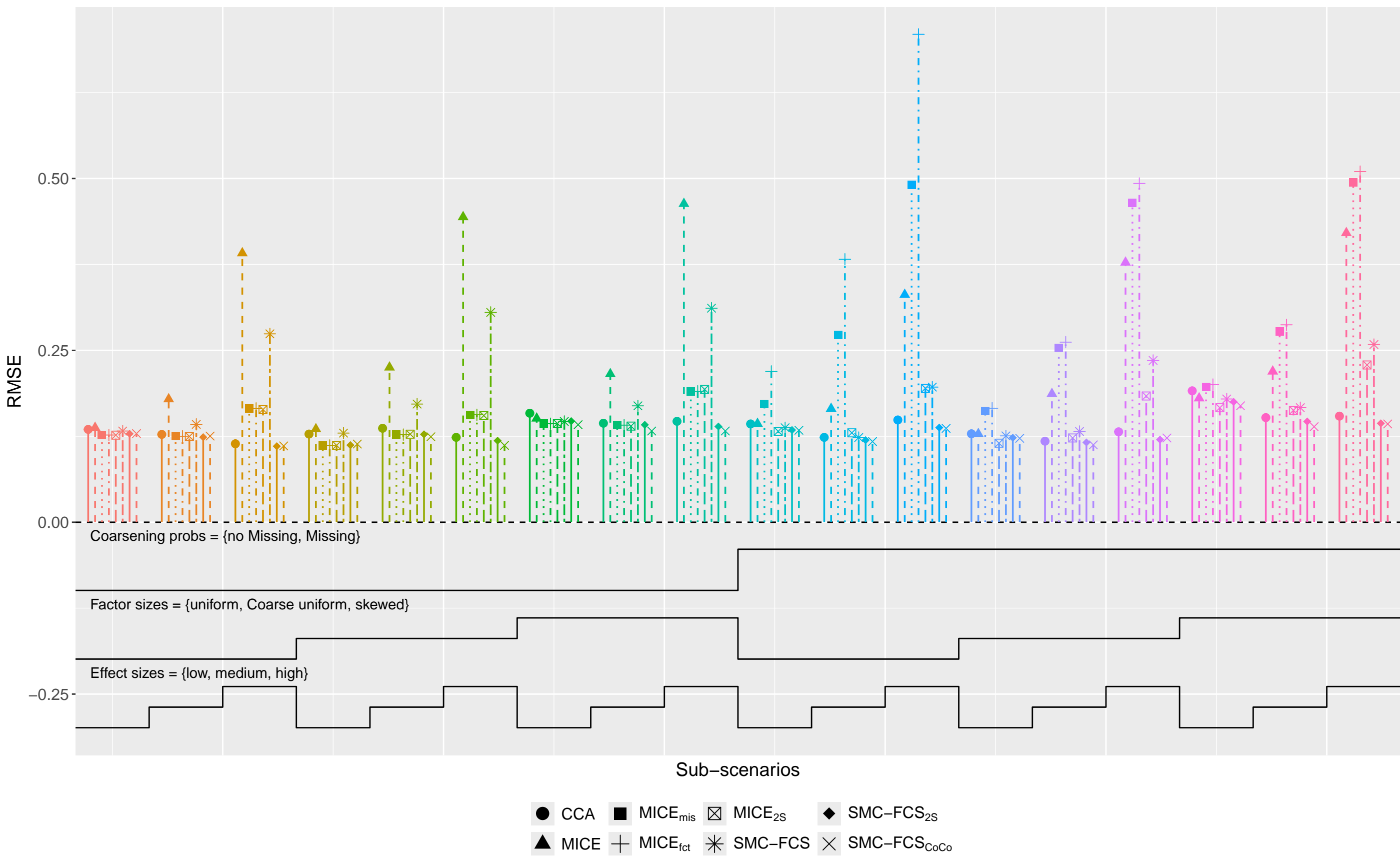

Supplement: Supplementary file 1 — Figures S1–S5, Supporting Information. [file SIM-44-0-s001.zip › Figure_S5C_surv_RMSE_Xc.pdf]

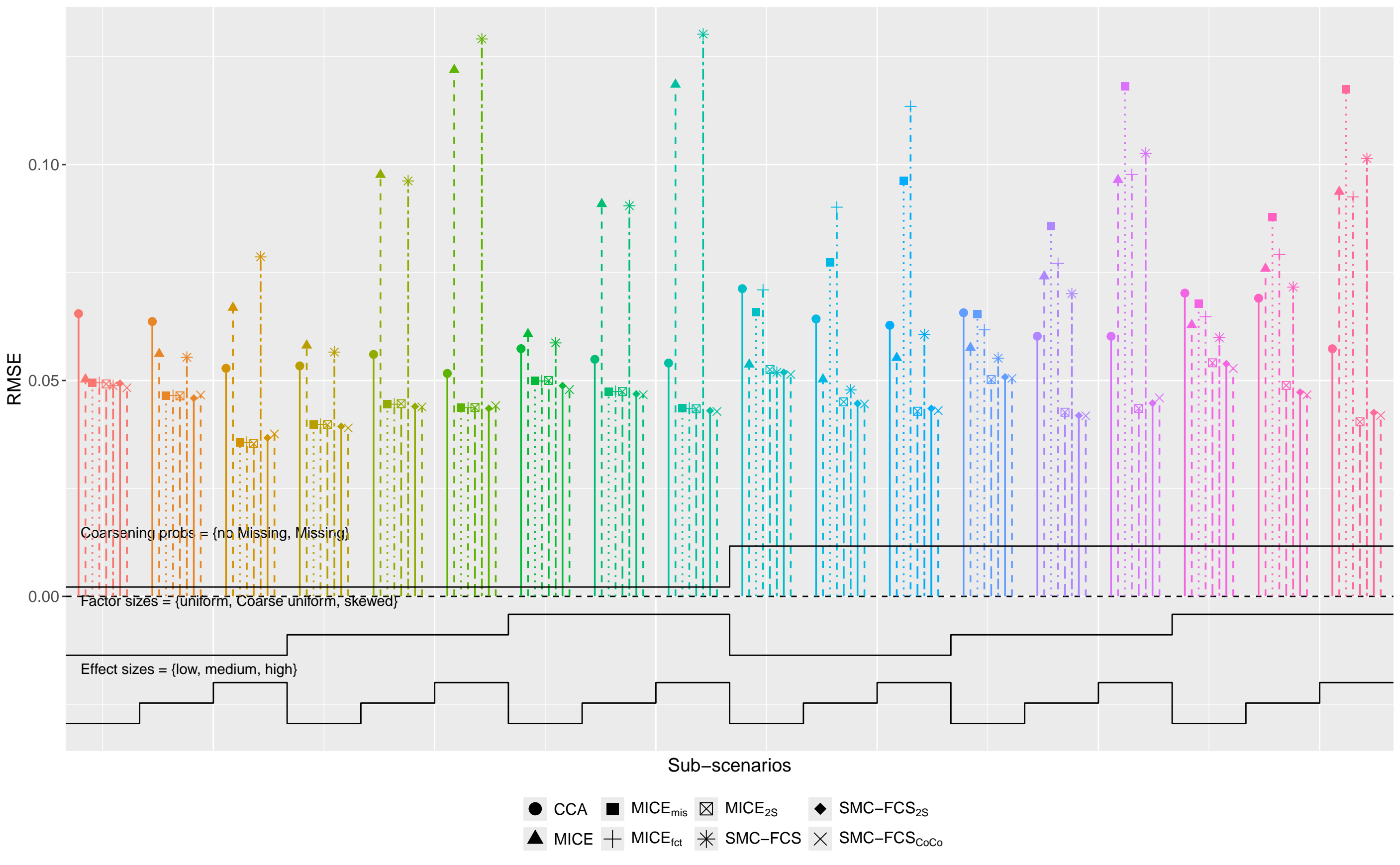

Supplement: Supplementary file 1 — Figures S1–S5, Supporting Information. [file SIM-44-0-s001.zip › Figure_S5D_surv_RMSE_Z1.pdf]

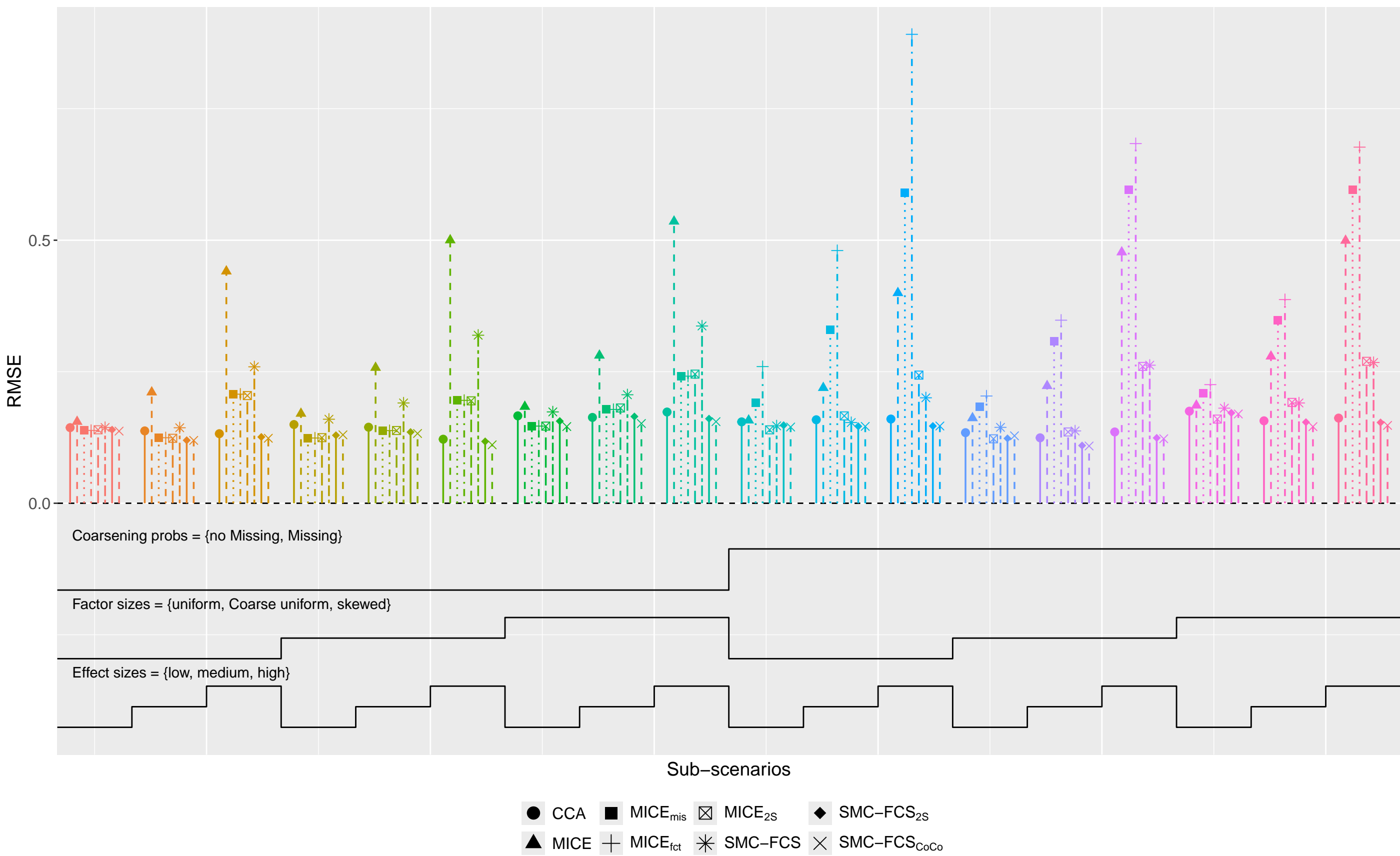

Supplement: Supplementary file 1 — Figures S1–S5, Supporting Information. [file SIM-44-0-s001.zip › Figure_S5E_surv_RMSE_Xc.pdf]

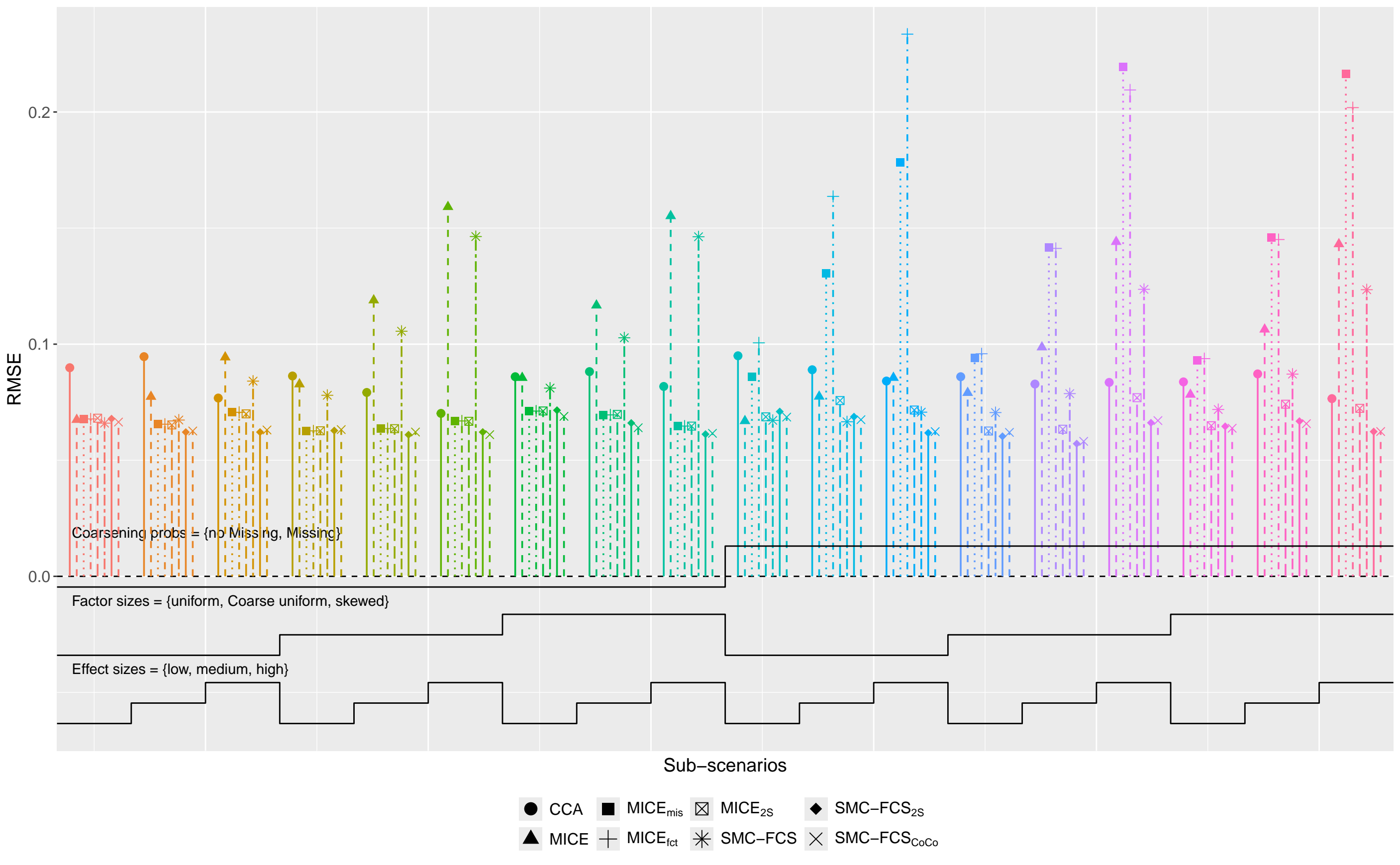

Supplement: Supplementary file 1 — Figures S1–S5, Supporting Information. [file SIM-44-0-s001.zip › Figure_S5F_surv_RMSE_Z1.pdf]
